# Blood Biomarkers for Traumatic Brain Injury: A Quantitative Assessment of Diagnostic and Prognostic Accuracy

**DOI:** 10.3389/fneur.2019.00446

**Published:** 2019-04-26

**Authors:** Zoe S. Gan, Sherman C. Stein, Randel Swanson, Shaobo Guan, Lizette Garcia, Devanshi Mehta, Douglas H. Smith

**Affiliations:** ^1^University of North Carolina School of Medicine, Chapel Hill, NC, United States; ^2^Department of Neurosurgery, Perelman School of Medicine, University of Pennsylvania, Philadelphia, PA, United States; ^3^Department of Physical Medicine and Rehabilitation, Perelman School of Medicine, University of Pennsylvania, Philadelphia, PA, United States; ^4^Rehabilitation Medicine Service, Corporal Michael J. Crescenz VA Medical Center, Philadelphia, PA, United States; ^5^Center for Neurotrauma, Neurodegeneration and Restoration, Corporal Michael J. Crescenz VA Medical Center, Philadelphia, PA, United States; ^6^Department of Neurosurgery, Perelman School of Medicine, Center for Brain Injury and Repair, University of Pennsylvania, Philadelphia, PA, United States

**Keywords:** traumatic brain injury, TBI, concussion, diagnosis, prognosis, biomarker, biomarkers

## Abstract

Blood biomarkers have been explored for their potential to provide objective measures in the assessment of traumatic brain injury (TBI). However, it is not clear which biomarkers are best for diagnosis and prognosis in different severities of TBI. Here, we compare existing studies on the discriminative abilities of serum biomarkers for four commonly studied clinical situations: detecting concussion, predicting intracranial damage after mild TBI (mTBI), predicting delayed recovery after mTBI, and predicting adverse outcome after severe TBI (sTBI). We conducted a literature search of publications on biomarkers in TBI published up until July 2018. Operating characteristics were pooled for each biomarker for comparison. For detecting concussion, 4 biomarker panels and creatine kinase B type had excellent discriminative ability. For detecting intracranial injury and the need for a head CT scan after mTBI, 2 biomarker panels, and hyperphosphorylated tau had excellent operating characteristics. For predicting delayed recovery after mTBI, top candidates included calpain-derived αII-spectrin N-terminal fragment, tau A, neurofilament light, and ghrelin. For predicting adverse outcome following sTBI, no biomarker had excellent performance, but several had good performance, including markers of coagulation and inflammation, structural proteins in the brain, and proteins involved in homeostasis. The highest-performing biomarkers in each of these categories may provide insight into the pathophysiologies underlying mild and severe TBI. With further study, these biomarkers have the potential to be used alongside clinical and radiological data to improve TBI diagnostics, prognostics, and evidence-based medical management.

## Introduction

Traumatic brain injury (TBI) is a common cause of disability and mortality in the US (1) and worldwide (2). Pathological responses to TBI in the CNS include structural and metabolic changes, as well as excitotoxicity, neuroinflammation, and cell death (3, 4). Fluid biomarkers that may track these injury and inflammatory processes have been explored for their potential to provide objective measures in TBI assessment. However, at present there are limited clinical guidelines available regarding the use of biomarkers in both the diagnosis of TBI and outcome prediction following TBI. To inform future guideline formulation, it is critical to distinguish between different clinical situations for biomarker use in TBI, such as detection of concussion, prediction of positive and negative head computed tomography (CT) findings, and prediction of outcome for different TBI severities. This allows for comparisons to determine which biomarkers may be used most appropriately to characterize different aspects of TBI.

The identification of TBI severity has become a contentious issue. Currently, inclusion in TBI clinical trials is primarily based on the Glasgow Coma Scale (GCS), which stratifies patients into categories of mild, moderate, and severe TBI. The GCS assesses consciousness and provides prognostic information, but it does not inform the underlying pathologies that may be targeted for therapy (5, 6). Furthermore, brain damage and persistent neurological symptoms can occur across the spectrum of TBI severity, limiting the use of GCS-determined injury severity to inform clinical management. Biomarkers in TBI have the potential to provide objective and quantitative information regarding the pathophysiologic mechanisms underlying observed neurological deficits. Such information may be more appropriate for guiding management than initial assessments of severity alone. Since the existing literature primarily focuses on applications of biomarkers in either suspected concussion, mild TBI (mTBI), or severe TBI (sTBI), we will discuss biomarker usage in these contexts.

Concussion is a clinical syndrome involving alteration in mental function induced by head rotational acceleration. This may be due to direct impact or unrestrained rapid head movements, such as in automotive crashes. Although there are over 30 official definitions of concussion, none include the underlying pathology. Missing from the literature have been objective measures to not only identify the underlying pathology associated with the given clinical symptoms, but also to indicate prognosis in long-term survival. Indeed, current practices in forming an opinion of concussion involve symptom reports, neurocognitive testing, and balance testing, all of which have elements of subjectivity and questionable reliability (7). While such information generally reflects functional status, it does not identify any underlying processes that may have prognostic or therapeutic consequences. Furthermore, because patients with concussion typically present with negative head CT findings, there is a potential role for blood-based biomarkers to provide objective information regarding the presence of concussion, based on an underlying pathology. This information could inform management decisions regarding resumption of activities for both athletes and non-athletes alike.

Blood-based biomarkers have utility far beyond a simple detection of concussion by elucidating specific aspects of the injury that could drive individual patient management. For example, biomarkers may aid in determining whether a mTBI patient presenting to the emergency department requires a CT scan to identify intracranial pathology. The clinical outcome for a missed epidural hematoma in which the patient is either discharged or admitted for routine observation is catastrophic; 25% are left severely impaired or dead (8). The Canadian CT Head Rule (9) and related clinical decision instruments achieve high sensitivities in predicting the need for CT scans in mild TBI cases. However, they do this at specificities of only 30–50% (10). Adding a blood biomarker to clinical evaluation may be useful to improve specificity without sacrificing sensitivity, as recently suggested (11). In addition, given concern about radiation exposure from head CT scans in concussion cases, particularly in pediatric populations, identification of patients who would be best assessed with neuroimaging is crucial. Thus, the use of both sensitive and specific biomarkers may serve as cost-effective tools to aid in acute assessment, especially in the absence of risk factors for intracranial injury (12). S-100B, an astroglial protein, has been the most extensively studied biomarker for TBI thus far and has been incorporated into some clinical guidelines for CT scans (13, 14). However, S-100B is not CNS-specific (15, 16) and has shown inconsistent predictive capacity in the outcome of mild TBI (17, 18). Given that several other promising biomarkers have also been investigated in this context, it is important to evaluate and compare the discriminative abilities of S-100B with other candidate blood-based biomarkers for future use.

Blood biomarkers also have the potential to help predict unfavorable outcomes across the spectrum of TBI severity. Outcome predication is difficult; in mTBI, existing prognostic models performed poorly in an external validation study (19). Identifying biomarkers that best predict delayed recovery or persistent neurological symptoms following mTBI would help with the direction of resources toward patients who may benefit most from additional rehabilitation or prolonged observation. In sTBI, poorer outcome has often been associated with a low GCS score (20). However, factors such as intoxication or endotracheal intubation may make it difficult to assess GCS reliably in the acute setting (21, 22). The addition of laboratory parameters to head CT and admission characteristics have improved prognostic models (23). Thus, prognostic biomarkers in sTBI could help determine whether patients are likely to benefit from intensive treatment. Several candidate biomarkers that correlate with various pathologies of mild and severe TBI have been studied (24), but their relative prognostic abilities remain unclear.

Existing reviews on biomarkers in TBI have provided valuable insight into the pathologic correlates of biomarkers, as well as how biomarkers may be used for diagnosis and prognosis (25–31). However, there has been no previous quantitative comparison of the literature regarding biomarkers' discriminative abilities in specific clinical situations. Here, we compare existing studies on the discriminative abilities of serum biomarkers for four commonly studied clinical situations: detecting concussion, predicting intracranial damage after mTBI, predicting delayed recovery after mTBI, and predicting adverse outcome after sTBI.

## Materials and Methods

### Categories

There has been substantial confusion about the role of blood-based biomarkers in TBI. Therefore, we chose four scenarios in which blood biomarkers might be considered most helpful:

1. To document whether a concussion has occurred, especially when the history is unclear. This might be most useful for professional athletes and military service members, for whom decisions to return to play or to combat could have serious consequences. This assessment relied on individual authors to define concussion, as no single gold standard definition exists.
2. To predict intracranial damage after mTBI (GCS 13–15). This could help decide whether or not a CT scan is indicated to identify occult intracranial lesions with potentially catastrophic consequences.
3. To predict delayed recovery after mTBI (GCS 13–15). This might help direct early rehabilitation therapy to patients at risk of a poor outcome. It could also serve to select these patients as clinical study subjects to evaluate treatment efficacy. This assessment allowed individual authors to define recovery given the variety of clinically relevant endpoints.
4. To predict outcome after sTBI (GCS ≤8). This might help alert the healthcare team in cases in which intensive treatment is either helpful or futile, as well as providing prognostic information to the patient's family.

Although several other potential uses of biomarkers have been suggested, we thought these four categories were the most useful clinically and had been covered most thoroughly in the literature. We omitted analysis of publications in which the outcome categories did not conform to the four categories or were unclear. We also elected to limit our analysis to biomarkers measured in peripheral blood and exclude reports of measurements done on CSF, brain tissue, urine, etc. Studies which reported results obtained too long after injury to be of predictive value were not included in the analysis. Cutoff points were 48 h for Category 1, 24 h for Category 2, 72 h for Category 3 and 7 days for Category 4.

### Literature Search

We conducted a search of Medline, Embase and the Cochrane library for reports of biomarkers of TBI published in English up to July 2018. The search strategy was limited to articles which included the medical subject headings of both “head injury” and “brain injury,” along with either “biomarker(s)” or “marker(s)” in the text. Additional articles were obtained from the bibliographies of selected reports and from the “Similar articles” feature of PubMed. Abstracts limited to animal studies or to samples other than blood were excluded. All other articles were downloaded and reviewed by at least two authors (SS, ZG, KG, DM, LG).

### Data Management and Analysis

Data abstracted from each report included TBI category, biomarker(s) measured, time(s) after injury, number of observations, cut point (point dividing positive from negative tests), sensitivity, specificity, area under the receiver operating curve (AUC) (32), any additional features reported (injury mechanism, age of subjects, outcome measured, etc.). If the TBI cohort of a given study was of mixed severity, and at least 70% of the patients met the severity criteria for a certain category, then the study was assigned to that category. Series which included adverse GOS scores were included in the severe injury category, even if fewer than 70% of reported cases had sTBI. For each set of observations, we calculated the AUC if not already provided. We also calculated the Youden J-statistic (33), another measure of diagnostic accuracy. A detailed discussion of diagnostic accuracy is given in the Supplementary Appendix.

If multiple reports dealt with the use of the same biomarkers to predict the same outcome, we pooled the data to obtain a single measure. For the AUC, we used a random-effects, inverse variance-weighted meta-analytic model to pool values (34). Since only the maximum J-statistic is used to report on a series of sensitivity/specificity values, we chose only the highest J-statistic measurement for each biomarker. We compared reported biomarkers with how well they predicted outcomes in a given category. We used a previously proposed semi-quantitative scale (35) to rate the accuracy of tests from their AUC's. An AUC above 0.9 is considered excellent, with decreasing intervals of 0.1 through “good,” “fair” and “poor.” An AUC below 0.6 is graded a “fail.”

A number of studies reporting mean biomarker levels were excluded if it was not possible to calculate operating characteristics from the published data. Other reports were excluded for reporting biomarker levels only as combinations of multiple markers or trajectories of a single marker over time.

## Results

### Literature Search

Our search yielded 2,015 publications, of which 1,034 abstracts were omitted as being unlikely to provide useful data. The remaining 981 articles were downloaded and reviewed. We excluded 233 reviews, editorials, letters to the editor, duplicate reports and other publications containing no original data. Also excluded were 162 case series limited to tissue other than blood (CSF, brain tissue, etc.), 40 reports containing fewer than 10 observations and 346 reports not relevant to the four outlined scenarios or from which operating characteristics could not be calculated. Included for analysis were 200 publications, encompassing a total of 61,722 observations.

**Figure 1:**
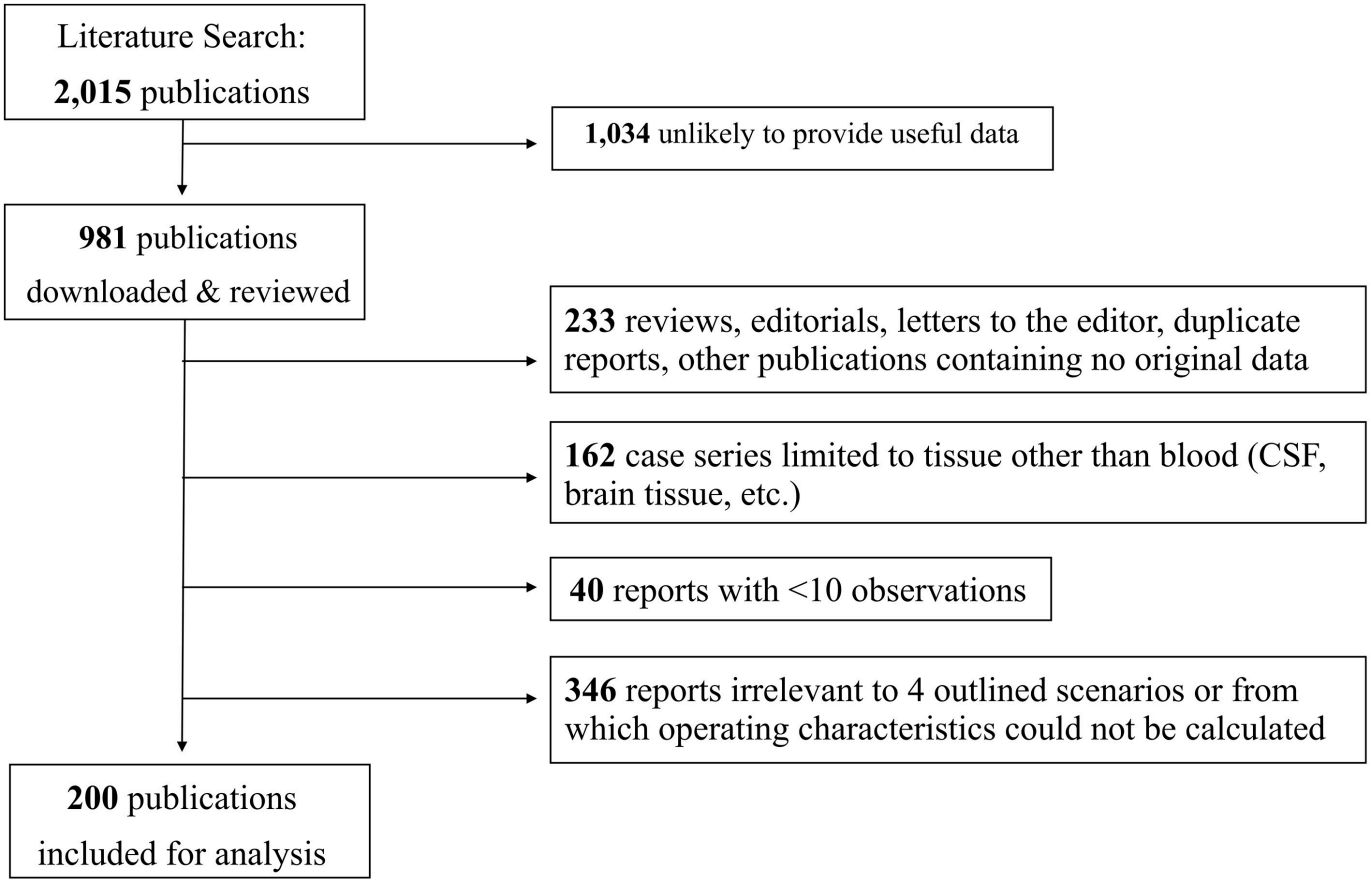
Flow chart of the study selection process.

The flow chart of the study selection process is shown in Figure 1. Included studies are listed by category in Table S1 (Supplementary Appendix), along with the biomarker tested and the number of observations. It should be noted that several reports are listed more than once, owing to their reporting on multiple biomarkers or multiple scenarios. An alphabetical list of abbreviations for biomarkers reported in the tables and the remainder of the manuscript is shown in Table 1.

**Table 1:** Abbreviations used for biomarkers.

**Abbreviation**	**Full name**
A-beta-42	Amyloid beta peptide
A-Tau	Tau-protein A
BDNF	Brain-derived neurotrophic factor
BMX	Tyrosine kinase
CKBB	Creatine kinase B type
CRP	C-reactive protein
C-Tau	Tau-protein C
FDP	Fibrin degradation products
GFAP/GFAP-BDP	Glial fibrillary acidic protein (breakdown products)
GM-CSF	Granulocyte-macrophage colony-stimulating factor
GSH	Glutathione
H-FABP	Heart-fatty acidic binding protein
HMGB1	High-mobility group box 1 gene
Hsp70	Heat shock protein
ICAM-1,-5	Intercellular adhesion molecule-1 and−5
icORP	Capacity for induced oxidative stress
IL-1beta, -6, -10	Interleukins
INR	International normalized ratio
LGALS3	Galectin 3
MBL	Mannose-binding lectin
MBP	Myelin basic protein
MCP-1	Monocyte chemoattractant protein
MDA-LDL	Malondialdehyde modified low density lipoprotein
MIF	Macrophage migration inhibitory factor
MMP-2 -,9	Matrix metalloproteinase-2 and−9
MT3	Metallothionein 3
NCAM	Neuron cell adhesion molecule
NF-H	Hyperphosphorylated neurofilament
NFL	Neurofilament light
NFM	Neurofilament medium
NRGN	Neurogranin
NSE	Neuron-specific enolase
OCLN	Occludin
pNF-H	Phosphorylated neurofilament heavy protein
PRDX-6	Peroxiredoxin
PTT	Partial thromboplastin time
P-Tau	Hyperphosphorylated tau
RDW	Red cell distribution width
S-100A1B, -A12, -B	S-100 calcium-binding proteins
SCUBE1	Signal peptide-cub-egf domain-containing protein-1
SNTF	Calpain-derived αII-spectrin N-terminal fragment
SuPAR	Soluble urokinase plasminogen activator receptor
sVCAM-1	Soluble vascular cell adhesion molecule-1
TAC	Total antioxidant capacity
TIMP-1	Tissue inhibitor of matrix metalloproteinase 1
T-Tau	Total tau
UCH-L1	Ubiquitin C-terminal hydrolase
VWF	Von Willebrand factor

### Analyses

#### Category 1 (Document Concussion)

There were 9 unique publications, documenting 15 biomarkers and containing a total of 946 observations. Several but not all authors defined concussion based on the 2012 Concussion in Sports Group guidelines (36) or the 2011 Team Physician Consensus Statement (37). Table 2 shows the pooled values for AUC and the maximum J-statistic obtained for each. Four biomarker panels (copeptin, galectin-3, and MMP-9; GFAP and UCH-L1; 10 metabolites; and 17 metabolites) are in the “excellent” range (AUC≥0.9). The AUC for copeptin, CKBB, and a 10-metabolite panel are also “excellent,” and 3 other biomarkers, galectin 3, MMP-9, and occludin rate as “good” (AUC = 0.80
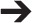
0.89). However, the observations are few, and no study has been independently verified.

**Table 2:** Presence of Concussion.

**Biomarker**	**#Reports**	**#Observations**	**Pooled AUC**	**Maximum J-statistic**
CKBB	1	18	**0.902**	0.602
copeptin	1	55	**0.922**	0.766
GFAP	2	238	0.533	0.030
LGALS3	1	55	**0.849**	0.508
MMP9	1	55	**0.846**	0.655
OCLN	1	55	**0.836**	0.562
panel (10 metabolites)	1	10	**0.976**	0.778
panel (17 metabolites)	1	29	**0.910**	0.76
panel(copeptin, LGALS3, MMP-9)	1	55	**0.968**	0.79
panel(GFAP, UCH-L1)	1	206	**0.940**	
panel(UCH-L1, S-100B)	1	32	0.750	
S-100B	2	108	0.680	0.441
SNTF	1	28	0.760	0.550
T-Tau	1	28	0.740	0.303
A-Tau	1	28	0.750	0.500
C-Tau	1	28	0.711	0.422
Ubiquitin	1	206	0.670	
UCH-L1	1	32	0.740	0.500

*Best results are indicated in boldface. Blank cells = either no data reported in original publication, or pooled AUC was considered too low (below “good” range, <0.80) to calculate J-statistic. See Table 1 for abbreviations*.

#### Category 2 (Need for CT Scan After mTBI)

There are 56 publications and 23,316 observations of 24 biomarkers in this category. As shown in Table 3, a single report shows excellent operating characteristics for two panels of biomarkers (MMP-2, CRP, and CKBB; UCH-L1 and GFAP), as well as for phospho tau (P-tau) and its ratio with total tau (P-tau/T-tau ratio). The UCH-L1/GFAP panel and P-tau also have excellent J-statistics. The AUC values for GFAP/GFAP-BDP and D-Dimer are in the “good” range; the excellent J-statistic for GFAP/GFAP-BDP is aided by a high specificity. S-100B protein, the most studied biomarker in this category, performs only in the fair category (AUC = 0.70
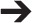
0.79).

**Table 3:** Mild TBI—need for CT scan.

**Biomarker**	**#Reports**	**#Observations**	**Pooled AUC**	**Maximum J-statistic**
A-beta-42	1	46	0.689	
BDNF	1	159	0.670	0.839
CKBB	1	92	0.714	
CRP	1	92	0.698	
d-Dimer	2	93	**0.890**	0.669
GFAP/GFAP-BDP	16	2040	**0.831**	0.936
GM-CSF	1	92	0.432	
H-FABP	2	264	0.641	0.293
IL-10	1	133	0.646	0.318
MDA-LDL	1	92	0.497	
MMP-2	1	92	0.616	
MT3	1	306	0.590	
NF-H	1	68	0.717	0.575
NFM	1	52	0.605	0.211
NRGN	1	494	0.510	
NSE	5	844	0.798	0.690
panel(MMP-2, CRP, CKBB)	1	110	**0.964**	0.7190
panel(UCH-L1, GFAP)	1	1947	**0.986**	0.9710
S-100B	30	8464	0.723	0.580
P-Tau	2	350	**0.921**	0.944
T-Tau	6	176	0.666	0.440
P-Tau/T-Tau ratio	2	350	**0.923**	0.816
Ubiquitin	2	302	0.710	0.210
UCH-L1	5	3108	0.700	0.470

*Best results are indicated in boldface. Blank cells = either no data reported in original publication, or pooled AUC was considered too low (below “good” range, <0.80) to calculate J-statistic. See Table 1 for abbreviations*.

#### Category 3 (Delayed Recovery After mTBI)

There are 44 publications reporting results of 29 biomarkers in 13,291 observations. Most but not all authors defined delayed recovery as post-concussive syndrome (PCS) at various time points after injury (notably, there is current debate regarding the term “PCS”). As shown in Table 4, small studies suggest that ghrelin, glucose, NFL, SNTF, and A-tau have AUC values in the “good” range and show promise for predicting mTBI patients who can be expected to suffer prolonged neurobehavioral or post-concussive symptoms. More commonly-studied biomarkers, such as GFAP, S-100B, NSE, and UCH-L1, have fair to poor discriminating ability.

**Table 4:** Mild TBI—delayed recovery.

**Biomarker**	**#Reports**	**#Observations**	**Pooled AUC**	**Maximum J-statistic**
BDNF	1	299	0.585	
BMX	1	63	0.760	0.400
CRP	1	846	0.615	0.330
GFAP	17	1959	0.716	0.850
Ghrelin	1	118	**0.829**	0.659
GSH	1	88	0.773	0.514
ICAM-1	1	118	0.485	
IL-6	1	118	0.535	
IL-8	1	118	0.615	
NCAM	1	118	0.614	
Neuroglobin	1	34	0.682	
NFL	1	35	**0.82**	0.79
Nogo-A	1	34	0.754	
NSE	6	543	0.685	0.691
pNF-H	1	118	0.614	
S-100B	24	2800	0.691	0.810
E-selectin	1	118	0.600	
SNTF	2	73	**0.863**	0.750
regulatory T cells	1	40	0.592	
Testosterone	1	181	0.684	0.786
VCAM-1	2	186	0.654	0.481
A-Tau	1	56	**0.87**	0.77
C-Tau	1	56	0.59	0.28
P-Tau	1	134	0.663	0.350
T-Tau	5	335	0.640	0.863
P-Tau/T-Tau ratio	1	134	0.658	0.300
UCH-L1	7	3158	0.787	0.740

*Best results are indicated in boldface. Blank cells = either no data reported in original publication, or pooled AUC was considered too low (below “good” range, <0.80) to calculate J-statistic. See **Table 1** for abbreviations*.

#### Category 4 (Poor Outcome After sTBI)

In this category, 85 publications reported 23,442 observations of 59 different biomarkers. As shown in Table 5, several biomarkers had “good” ability to predict death, severe disability or other adverse outcomes after sTBI. They include ceruloplasmin, copeptin, D-Dimer, ficolin-3, galectin-3, gelsolin, H-FABP, HMGB1, icORP, IL-1beta, −6 and −8, leptin, MBL, MBP, MIF, NFM, periostin, RDW, S100A12, SCUBE1, SuPAR, TAC, tenascin-C, thrombospondin-1, and T-tau. However, numbers of observations are small, and independent verification is lacking.

**Table 5:** Severe TBI—adverse outcomes.

**Biomarker**	**#Reports**	**#Observations**	**Pooled AUC**	**Maximum J-statistic**
Adiponectin	1	86	0.785	0.604
Base deficit	1	216	0.479	
BDNF	‘	170	0.482	
Caspase-Cleaved Cytokeratin-18	1	100	0.685	0.370
ceruloplasmin	1	20	**0.800**	0.600
Cholinesterase	1	188	0.381	0.519
Copeptin	4	422	**0.825**	0.635
Copper	1	20	0.795	0.590
D-Dimer	2	226	**0.819**	0.895
DNA	2	106	0.694	0.430
FDP	1	1266	0.755	0.426
Ferritin	1	69	0.585	0.170
Fibinogen	1	1266	0.712	0.382
Ficolin-3	1	384	**0.823**	0.619
Galectin-3	1	300	**0.808**	0.553
Gelsolin	2	322	**0.805**	0.679
GFAP	10	2448	0.749	0.800
H-FABP	1	49	**0.840**	0.680
HMGB1	1	106	**0.882**	0.657
Hsp70	1	20	0.750	0.500
ICAM-1	1	13	0.498	0.222
ICAM-5	1	170	0.544	
icORP	1	104	**0.870**	0.333
IL-1beta	1	28	**0.871**	0.800
IL-6	3	337	**0.840**	0.840
IL-8	1	20	**0.835**	0.67
IL-10	1	426	0.550	0.265
INR	1	1266	0.738	0.394
Leptin	1	284	**0.875**	0.649
MBL	1	244	**0.832**	0.562
MBP	2	127	**0.831**	0.875
MCP-1	1	170	0.677	
MDA	1	100	0.760	0.370
MIF	1	216	**0.817**	0.547
MMP-9	1	88	0.585	0.340
Nesfatin	1	300	0.786	0.487
NF-H	2	200	0.760	0.552
NFL	1	70	0.700	0.390
NFM	1	12	**0.857**	0.714
NSE	9	911	0.715	0.905
Periostin	1	130	**0.815**	0.506
Platelet count	1	1266	0.618	0.201
PRDX-6	1	170	0.524	
PTT	1	1266	0.748	0.410
RDW	1	122	0.693	0.611
S-100A1B	1	59	0.677	0.3
S100A12	1	306	**0.855**	0.630
S-100B	25	3712	0.762	0.880
SCUBE1	1	113	**0.831**	
Substance P	1	100	0.700	0.360
SuPAR	1	78	**0.801**	0.363
TAC	1	100	**0.830**	0.410
Tenascin-C	1	216	**0.827**	0.590
Thioredoxin	1	216	0.798	0.549
thrombospondin-1	1	402	**0.827**	0.619
TIMP-1	1	100	0.645	0.290
T-Tau	6	344	**0.818**	0.833
UCH-L1	5	195	0.696	0.54
VWF	1	44	0.660	0.32

*Best results are indicated in boldface. Blank cells = no data reported in original publication; pooled AUC was considered too low (below “good” range, <0.80) to calculate J-statistic. See Table 1 for abbreviations*.

## Discussion

**Figure 2:**
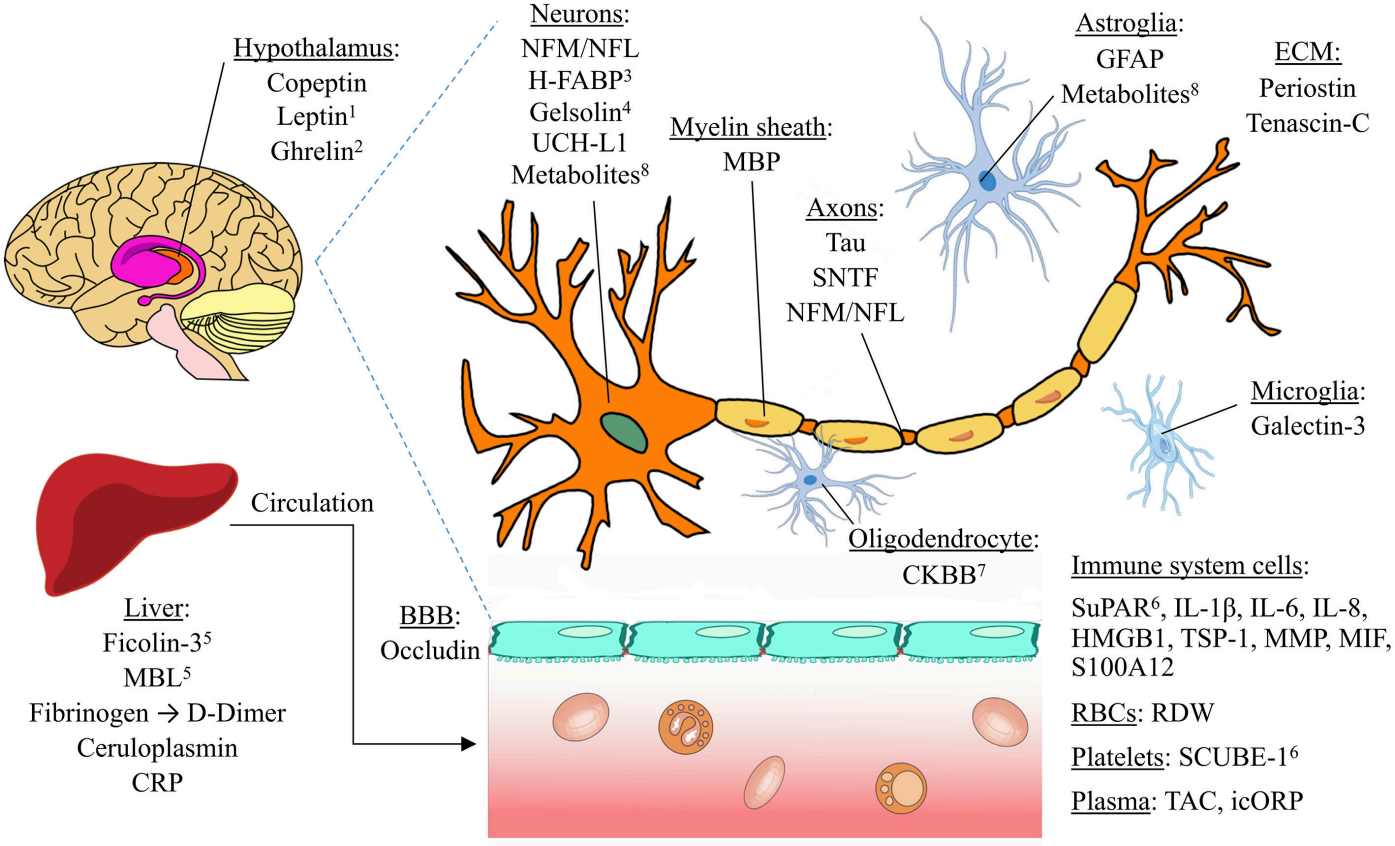
Anatomical locations of potential TBI biomarkers. The biomarkers included in this schematic all rated as “good” (AUC=0.80
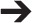
0.89) or better for any of the four clinical situations studied (detecting concussion, predicting intracranial damage after concussion, predicting delayed recovery after concussion, and predicting adverse outcome after severe TBI). Biomarkers with a pooled AUC <0.8 are not shown. ^1^Also found in adipose tissue; ^2^synthesized in cells of stomach and pancreas; may regulate HPA axis; ^3^found mostly in pons; ^4^also found extracellularly; ^5^lectin pathway of the complement system; ^6^also found in endothelial cells. BBB, blood brain barrier. ECM, Extracellular matrix. Image licensed under Creative Commons Attribution-ShareAlike 4.0 International license. https://creativecommons.org/licenses/by-sa/4.0/deed.en. See Supplementary Material for image credits and licensing.

We have identified leading candidate biomarkers potentially useful for four clinical purposes in TBI, as determined by the highest pooled AUC and J-statistic from the existing data. Figure 2 provides a visual overview of the candidate biomarkers' anatomical locations. These biomarkers have the potential to be used not as stand-alone diagnostic or prognostic tests, but rather alongside clinical and radiological data in the collective process of forming a clinical decision. In particular, in the absence of acute or chronic behavioral changes, excessive focus on biomarker values may lead to unnecessary testing with negative psychological and economic consequences. Blood biomarkers offer potentially valuable objective information that may augment rather than replace existing tools for clinical assessment and contribute to a holistic approach to management.

### Category 1 (Document Concussion)

Two single biomarkers had excellent operating characteristics (AUC>0.9) for documenting concussion. Copeptin, the C-terminal part of the arginine vasopressin (AVP) prohormone, is thought to reflect the hypothalamic pituitary adrenal axis activity as part of the stress response, and serum levels increase in proportion to TBI severity (38, 39). CKBB is an intracellular enzyme that catalyzes the phosphorylation of creatine to phosphocreatine as part of cellular energy homeostasis and is primarily found in oligodendrocytes, which may be due to large energy requirements in these cells (40). The good performance of these biomarkers suggests that both stress axis activation and cellular damage within specific brain areas are involved in the pathophysiology of concussion.

Three other biomarkers, galectin 3, MMP-9, and occludin, had good operating characteristics (AUC = 0.80–0.89) for detecting concussion based on a single study (ref. S17 in Supplementary Appendix) while the combination of all 3 yielded an excellent operating characteristic. Galectin-3, a beta–galactoside-binding lectin, was previously found to be expressed in activated microglia after diffuse axonal injury (DAI) (41). MMP-9, a matrix metalloprotease that is expressed in humans early after TBI (42), modifies the brain extracellular matrix and leads to cerebral edema and disruption of blood-brain barrier (BBB) integrity following TBI (43, 44). OCLN is a regulatory protein at the tight junctions of the BBB that correlates with increased resistance and decreased permeability of the BBB (45). While these findings identify osmotic dysregulation, BBB disruption, cerebral edema, and DAI as potential pathologic correlates of concussion, conclusions regarding clinical utility are limited by the relatively small sample size and lack of independent verification. Furthermore, concern has been raised about the authors' limited characterization of the control group and its subsequent impact on their conclusions (46).

The superior performance of other biomarker panels in this category reflects the multifaceted pathophysiology associated with concussion. These panels appear to successfully gather data about different mechanisms of injury to maximize sensitivity and specificity. The combination of GFAP and UCH-L1, two biomarkers thought to reflect focal mass lesions and diffuse injuries, respectively, also performed at the “excellent” level (ref. S11). However, the combination of UCH-L1 and S-100B only had fair performance, reflective of the poor individual performance of the nonspecific marker S-100B. Incorporation of higher-performing individual biomarkers, such as copeptin and CKBB, into panels may be useful to study in the future. Metabolite panels demonstrated also excellent operating characteristics; some metabolites are thought to reflect altered brain energy metabolism and mitochondrial dysfunction in TBI (47). However, the use of a metabolite panel is limited by variability of specific metabolites used across studies (refs. S10, S12). Given the limitations of these single, small studies, further verification is warranted to identify the best candidate serum biomarkers for a panel to objectively detect concussion.

### Category 2 (Need for CT Scan After mTBI)

It should be noted that several clinical decision rules are available to predict the need for CT scan in mild TBI. These rules have near 100% sensitivity. However, their specificities are low (10), resulting in roughly 50% negative CT scans in those patients predicted to need them. A recent report (48) demonstrated that GFAP and UCH-L1 levels were no higher in patients with mild TBI and negative CT scans than in patients with orthopedic but not head injuries. This suggests that low GFAP and/or UCH-L1 levels may be useful in reducing unnecessary CT scans for mild TBI.

While the current study shows that operating characteristics were good for GFAP and its breakdown products and poor for UCH-L1, the use of both biomarkers in combination had excellent discriminative ability for identifying CT-positive mTBI. Another biomarker panel including MMP-2, CRP, and CKBB also had excellent performance, reflecting mechanisms of generalized inflammation (CRP), local brain inflammation (MMP-2), and cell membrane damage (CKBB, previously discussed in Category 1) (ref. S65). Our analysis shows superior performance of these two biomarker panels to S-100B, the only biomarker with a low-level recommendation for determining the need for a CT scan following mTBI (13). Panels may thus provide a more holistic approach to detecting intracranial injury warranting a CT scan.

Two individual biomarkers performed slightly lower than the panels but still rated as “excellent” based on single studies. The superior discriminative ability of P-tau compared to the more commonly studied T-tau highlights the significance of tau hyperphosphorylation in brain tauopathy. D-dimer also had good performance in this category in a pediatric population (ref. S23), although there are additional clinical scenarios that may cause an elevated D-dimer unrelated to TBI, such as trauma and infection (49). In addition, the applicability of this finding to adults is unknown, and the sample size is small. Further research is warranted to confirm the results of these single studies.

### Category 3 (Delayed Recovery After mTBI)

A few less-studied biomarkers performed best for predicting delayed recovery following mTBI. Single studies demonstrated that SNTF, tau A, ghrelin, and NFL all had operating characteristics in the “good” range, outperforming more commonly-studied biomarkers such as GFAP, S100B, NSE and UCH-L1.

Three axon-associated proteins, calpain-derived αII-spectrin N-terminal fragment (SNTF), tau-A, and NFL, may be indicative of DAI, which is thought to be one of the most common pathological mechanisms accounting for long-term dysfunction in all severities of TBI (50–53). SNTF accumulates in damaged axons (54–56) following intra-axonal calcium overload and calpain-mediated proteolysis in stretch injury (57, 58). Furthermore, SNTF has been found in degenerating axons after TBI that were undetected by the gold standard marker of transport interruption, amyloid precursor protein (APP) (59). Tau protein may mediate DAI by regulating axon microtubule assembly; (60, 61) tau-A fragments in particular are easily detectable and quantifiable by standard ELISA, perhaps due to their small size, and subsequent ability to cross the BBB (62). NFL is predominantly expressed in subcortical axons and correlates with magnetic resonance diffusion tensor imaging parameters of DAI (63). The included studies found that elevated serum SNTF predicted failure to improve cognitive function at 3 months in CT-negative concussion patients (ref. S83), while tau A and NFL predicted late resolution of post-concussive symptoms in concussed professional ice hockey players (refs. S16, S82). Thus, these proteins may be mechanism-specific biomarkers for identifying patients at risk for persistent cognitive deficits following mTBI.

Ghrelin is an orexigenic peptide hormone that may be linked to stress-induced hypothalamic-pituitary axis (HPA) activation (64, 65) and cognitive dysfunction in neurodegenerative disease (66). In the included study, low values of ghrelin within the first few days following concussion were independently associated with three-month neurocognitive impairment (ref. S89). Thus, ghrelin may be a nonspecific prognostic indicator in mTBI to be used in conjunction with other brain-specific biomarkers such as SNTF.

Reliable biomarkers in this category have the potential to be used in conjunction with radiologic data as well as current predictors of worse outcome after mTBI, such as older age, lower level of education, and pre-existing psychiatric conditions (19). This could help identify patients at risk of persistent disability and the development of additional neurocognitive sequelae. However, as the results for tau-A, SNTF, NFL, and ghrelin were based on a handful of studies with relatively small sample sizes, these candidate biomarkers warrant further investigation regarding their prognostic abilities and rehabilitative implications in mTBI.

### Category 4 (Poor Outcome After sTBI)

For predicting mortality and poor outcome in sTBI, there were no biomarkers with operating characteristics in the “excellent” range. However, several biomarkers performed in the “good” range based on single studies, including markers of coagulation and inflammation, structural proteins in the brain, and regulatory proteins in normal homeostasis. The prognostic value of these downstream biological processes suggests that there may be potential for considering some TBIs as systemic rather than primarily localized disorders. Such a holistic approach could have significant implications for both acute and chronic treatments.

Serum biomarkers of coagulation with good ability to predict poor outcome in sTBI include D-Dimer, thrombospondin-1, and SCUBE1. D-dimer is thought to indicate TBI-induced coagulopathy (67–69) that largely occurs secondary to DIC and leads to further cerebral injury (70). Thrombospondin-1 is a thrombin-sensitive, anti-angiogenic factor (71, 72) whose expression is increased after intracerebral hemorrhage (73). SCUBE1 is released from endothelial cells and platelet alpha granules during platelet activation (74, 75). As coagulopathy in isolated TBI is associated with increased mortality (76, 77), D-Dimer, thrombospondin-1, and SCUBE1 could be important prognostic indicators in sTBI.

Several inflammatory markers with good operating characteristics were identified, including IL-1beta, IL-6, IL-8, HMGB1, ceruloplasmin, ficolin-3, macrophage inhibitory factor (MIF), MBL, galectin-3, S100A12, and SuPAR. HMGB1 had the highest pooled AUC in this category, based on a single study (ref. S165). HMGB1's high expression in the brain (78, 79) suggests that it may be useful for recognizing patients with critical inflammatory responses to brain injury that are associated with severe disability and death. While these markers of inflammation are not specific for brain-localized insults, they may contribute prognostic information by helping to characterize strong inflammatory responses to TBI that contribute to secondary brain injury (80) and ultimately poor outcome. The good performance of periostin and tenascin-C, two extracellular matrix proteins involved in various cell cycle processes including proliferation, migration, differentiation, and apoptosis, suggests that measures of cell turnover in response to injury may have prognostic value as well (refs. S105, S179).

Measurements of the capacity to endure oxidative stress also fared well. The brain is particularly susceptible to oxidative stress due to high oxygen consumption, limited neuron regeneration, and high levels of unsaturated fatty acids in membranes (81). In TBI, the release of reactive oxygen species (ROS) induces inflammation, compromise of the BBB, and cell death (82–85). Quantitation of antioxidants in the brain and the oxidative-reduction potential have subsequently been used to detect oxidative stress (81, 86, 87). The icORP measures the ability of a biological sample to endure an oxidative insult by using an oxidative current to deplete antioxidants in the sample (ref. S92), whereas TAC measures the capacity of antioxidants in a sample to prevent oxidation of a peroxidase substrate (ref. S126). These measures may indicate not only the extent of ongoing ROS-induced damage and inflammation, but also the limited ability of the body to deal with oxidative insults that translate into poor prognosis.

Structural proteins in the brain may also predict outcome as a result of brain-specific injury. High performers identified in this category were MBP, an abundant structural protein of the myelin sheath (88); tau protein, discussed earlier in Category 3 for its ability to predict delayed recovery after mTBI; and NFM, a type IV intermediate filament that contributes to neuron structure, as well as axonal structure and transport (89). Interestingly, the astroglial protein S100B, the most extensively studied biomarker in TBI, had a similar J-statistic but a lower pooled AUC when compared to biomarkers discussed here. Further prognostic studies on these biomarkers in multiple severe TBI populations, particularly on the less-studied MBP and NFM, may allow for better comparison with S-100B.

A handful of proteins involved in homeostatic functions also demonstrated good operating characteristics for predicting poor outcome. Copeptin, which was identified above as a promising marker for detecting concussion, also performed well in this category. This indicates that the degree of stress axis activation has prognostic implications in sTBI, although the prognostic value of copeptin is not limited to TBI (90). Gelsolin mediates cell shape changes & motility (91) and is decreased in acute tissue injury after trauma (92). Leptin, the “satiety hormone,” fared well in a pediatric population (ref. S121). It is secreted by adipose tissue (93, 94), is also expressed in the hypothalamus (95), and may play an important role in neuronal and glial maturation (96). H-FABP, which is involved in the intracellular traffic of fatty acids and other hydrophobic ligands, primarily reflects cardiac injury (97) but is also found in smaller concentrations in the brain (98, 99) and other tissues (100). Changes in these markers of osmoregulation, cell motility, energy homeostasis, and fatty acid trafficking may reflect systemic disturbances in sTBI that lead to poor prognosis.

Top-performing biomarkers in this category have the potential to inform which pathologic mechanisms may be most indicative of poor outcome after sTBI. While TBI pathophysiology is undoubtedly complex, making management decisions in this context challenging, the information provided by biomarkers may add value to existing prognostic models (101). The IMPACT (International Mission on Prognosis and Analysis of Clinical Trials) (102) and CRASH (Corticosteroid Randomization After Significant Head Injury) (103) models predict mortality and unfavorable outcome at 6 months after sTBI. Both models take into account age, GCS motor score, pupillary reactivity, and CT classification, and both have been externally validated with comparably reasonable discriminative ability (104). However, lower discriminative performance of these models in a different validation set (105) and at the individual level (106) perhaps indicates the need to update prognostic models to improve generalizability. Validation of promising markers identified in this analysis could potentially lead to the improvement of such models.

## Limitations

This study has a number of consequential limitations. Within each outcome category, the data exhibited considerable heterogeneity. Different patient populations, ages, definitions of outcome, and delays between injury and sampling all detract from the reliability of our findings. In particular, the numerous definitions of concussion and recovery in our included studies limit the strength of our conclusions in these categories. While this variability reflects the heterogeneous nature of these terms in clinical usage, we attempted to identify studies that fit into general categories of clinical interest. Furthermore, the small number of observations, often only a single small study, make statistical comparisons, and stratification (by age, time after injury, etc.) unreliable.

We omitted several otherwise-excellent studies in which levels of a particular biomarker were shown to be significantly associated with the presence of TBI sequelae of interest to us. However, in the absence of operating characteristics or individual subject measurements, we could not calculate how well the biomarker would predict the outcomes of interest. Other studies failed to separate head injuries of different severities or chose outcomes other than those of interest in this study. Biomarkers from tissues other than blood, combinations of biomarkers, and changes in their levels over time are potentially quite useful but beyond the scope of this study.

The developing field of anti-neuronal autoantibodies could be especially promising for predicting delayed recovery and chronic complications after TBI, across the spectrum of severity levels. An exponential increase in neuroimmunology research over the past decade has contributed to a significant shift in our understanding of anti-neuronal autoantibodies and led to the development of novel blood-based diagnostics for several neurological disorders (107–118). Following the landmark 2007 study that introduced anti-*N*-methyl-D-aspartate receptor (NMDAr) encephalitis (119), IgG autoantibodies against neuronal membrane targets have been implicated in the pathogenesis of various neurological disorders (119–128). Human studies investigating anti-neuronal autoantibodies present in the blood post-TBI have largely investigated the role of TBI-induced (adaptive) IgG autoantibodies, which appear ~4–6 days following TBI (129–132). However, it has recently shown that serum IgG autoantibodies are present in both human and animal serum, regardless of age, sex or disease state (133–136). The recent discovery that all human blood contains thousands of autoantibodies (133, 136) and that individual autoantibody profiles are influenced by the presence of disease (111, 113, 118, 123, 137) leads to the promising hypothesis that quantification of disease-specific changes in serum anti-neuronal autoantibody titer concentrations can serve as highly sensitive and specific biomarkers of persistent post-TBI neurodegeneration. Indeed, the discovery of non-invasive serum biomarkers such as autoantibody profiling which objectively demonstrate chronic post-TBI neurodegeneration would provide objective information to inform clinical trials for both mechanism discovery and therapeutic intervention.

Genetic variants have been increasingly studied to explain the variability in outcome following TBI. Many single nucleotide polymorphisms (SNPs), single nucleotide substitutions within a gene's coding or regulatory regions, have been identified for this purpose (138–140). In particular, SNPs in genes of proteins involved in dopamine availability and transmission have been targeted, as dopamine dysregulation after TBI is thought to contribute to chronic deficits in memory, attention, and executive function (141). SNPs in both catechol-O-methyltransferase (COMT) and ankyrin repeat and kinase domain-containing 1 (ANKK1) have been associated with a variety of cognitive impairments after predominantly mTBI (142–145), but this association is less clear in sTBI (146–149). A better understanding of which genes are implicated in the neurocognitive response to TBI may shed light on mechanisms of such injury and have both prognostic and therapeutic implications. Future studies will need to clarify the effects of age, gender, ethnicity, environment, and gene-gene interactions on the relationship between gene expression and brain function (150).

Finally, there are questions about the reliability of any blood biomarker as an indicator of brain injury severity. The integrity of the blood-brain barrier, as well as proteolytic degradation of some biomarkers in serum, could affect measured levels (26). Plog et al. hypothesize that the transport mechanisms, which they term the “glymphatic” system, may have a greater influence on biomarker levels than TBI severity itself (151). Thus, clinically relevant manipulations of this system, such as cisternotomy and sleep deprivation, could prevent accurate interpretation of serum biomarker levels. Peripheral surgical trauma also disrupts the BBB and leads to neuroinflammation (152). While comparing the discriminative abilities of CSF biomarkers may bypass these challenges, there exists much more data on blood biomarkers due in part to the ease and convenience with which they may be collected in a variety of settings. Due to these limitations, it must be emphasized that blood biomarkers have value not as isolated diagnostic tests, but rather as adjuncts to clinical, radiological, and other diagnostic information.

## Conclusion

We have reviewed the literature and identified blood biomarkers with the highest discriminative abilities as determined by operating characteristics in four commonly encountered clinical situations: diagnosing concussion, predicting the need for a CT scan after mTBI, predicting delayed recovery after mTBI, and predicting poor outcome after sTBI. The top performers in each category may provide insight into pathogenic mechanisms of TBI that most influence the measured endpoint. Nonetheless, many challenges remain before these biomarkers can be incorporated into clinical practice. In particular, it remains unclear whether a large panel of biomarkers in addition to clinical assessment will be sufficient to first stratify patients into categories of TBI before more specific biomarker assessments are applied. Alternatively, in the age of precision medicine, biomarker assessment may be tailored to individual patients. Ideally, pre-clinical development will help refine approaches for clinical application.

## Author Contributions

ZG, SG, LG, DM, and SS contributed to the acquisition, analysis, and interpretation of the data. SS, RS, and DS contributed to study conception and design. ZG, RS, and SS drafted the work, and all other authors (SG, LG, DM, DS) revised it critically for important intellectual content. All authors (ZG, SS, RS, SG, LG, DM, DS) provide approval for publication of the content and agree to be accountable for all aspects of the work in ensuring that questions related to the accuracy or integrity of any part of the work are appropriately investigated and resolved.

### Conflict of Interest Statement

The authors declare that the research was conducted in the absence of any commercial or financial relationships that could be construed as a potential conflict of interest.
