# Supplementary material for: Blood Biomarkers for Traumatic Brain Injury: A Quantitative Assessment of Diagnostic and Prognostic Accuracy
Source: Front Neurol. 2019 Apr 26;10:446. doi: 10.3389/fneur.2019.00446 (PMC6498532; doi:10.3389/fneur.2019.00446)
Supplement: Supplementary file 1 [file Data_Sheet_1.docx]

**Supplementary Appendix**

Literature search:

Table S1 illustrates the publications which provided data used in this analysis. For each category, the articles used list the first author, the biomarkers measured and the number of observations reported. The latter may not equal the number of cases, as many subjects had observations at multiple time points and/or multiple cut points.

Diagnostic Accuracy

The purpose of any diagnostic test is to identify those who have or are likely to develop a specific medical condition and to rule out those who do not. The ability of a test to do this is loosely termed “accuracy,” a term which is imprecise. There are several quantitative approaches to measuring diagnostic accuracy, some of which are discussed below. The interested reader is referred to an excellent general discussion of the subject and its role in medical decision making.[^1^](#_ENREF_1)

*Operating characteristics*

Figure S1 illustrates the performance of a theoretical test on 15 subjects, 5 of whom have the disease of interest. The test is measured in arbitrary units. If the test results are above a certain number of units, the test is considered positive, otherwise negative. The level used to separate positive from negative is termed the “cut point.” Figure S1 shows how many cases fall above and below seven different cut points. Figure S2 shows how one calculates the test’s successes and failures at a particular cut point. In the ideal test, all diseased subjects would test positive (true positives), and all healthy subjects would test negative (true negative). For example, using a cut point of 3 units in the example, 4 of 5 diseased subjects would test positive, 1 would test negative (false negative). At the same cut point, 6 of the 10 non-diseased subjects test negative, 4 positive (false positive).

Using these numbers, we can calculate a test’s sensitivity (true positive divided by all diseased) and specificity (true negative divided by all non-diseased). Each cut point yields a different combination of sensitivity and specificity. As is evident from Figure S1, the true positive rate (sensitivity) of the test falls as the cut point increases. Conversely, the true negative rate (specificity) increases with a rising cut point. Table S2 shows sensitivity and specificity associated with each cut point. The best combination of the two (the greatest sensitivity without sacrificing specificity) is called the “optimal operating point” (OOP). Determining the OOP is discussed below.

*Receiver Operating Characteristic (ROC) curves*

As is evident from Table S2, as sensitivity rises, specificity falls. Put another way, as the sensitivity of a given test increases, true positive rate increases, as does false positive rate. The latter is equivalent to 1 minus specificity, and the combination at each cut point can be plotted. The result, shown in Figure S3, is known as the receiver operating characteristic (ROC) curve. At a sensitivity of zero, both true positive and false positive rates = zero. At the opposite extreme, both = 1.

The shaded area under the curve (AUC) is widely considered a good measure of diagnostic accuracy. With perfect sensitivity and specificity, the AUC will be 1. An AUC of 0.5 is equivalent to flipping a coin to determine diagnosis. The AUC can be calculated from the areas of its composite polygons. For our example, the AUC = 0.715. A more complete explanation of ROC curves is given by Linden.[^2^](#_ENREF_2) A semi-quantitative scale has been proposed[^3^](#_ENREF_3) to rate the accuracy of tests from their AUC’s. An AUC above 0.9 is considered excellent, with decreasing intervals of 0.1 through “good,” “fair” and “poor.” An AUC below 0.6 is graded a “fail.”

*Youden’s J-statistic*

This is a way to rate a diagnostic test and also to approximate its OOP.[^4^](#_ENREF_4) For each cut point, the sensitivity and specificity are summed, and 1 is subtracted. The resulting value is the J-statistic for that cut-point and that test. The highest value obtained using this method with a given dataset is considered the optimal J-statistic for that test. Table S2 shows the calculations for our example. In cases where sensitivity and specificity were not provided and AUC was considered good or excellent, we calculated the J-statistic manually from the ROC curve. Where multiple publications were available for the same biomarker and scenario, we report only the maximum J-statistic for that biomarker and scenario.

Accuracy vs. discrimination

What we are describing as a test’s “accuracy” is really its ability to discriminate between the diseased and non-diseased state. Choosing a cut point or OOP which maximizes the combination of sensitivity and specificity assumes that both are of equal importance. However, this is not always the case. If, for example, a missed case of disease (false negative) has more harmful consequences that a missed case of non-disease (false positive), The OOP must shift toward greater sensitivity, even with a loss of specificity. The “cost of errors” can be calculated and used to determine a more accurate OOP. Figure S4 shows ROC curves of two different tests for the same disease. They have identical AUC’s and J-statistics. The superior test is the one in which errors are less costly, both in terms of patient outcomes and costs.

**Table S1: Publications Used in this Study**

| **First Author** | | **Number of Observations** | **Blood Biomarker** |
| --- | --- | --- | --- |
| **Rule out Concussion** | |  |  |
|  | Daley, M[^10^](#_ENREF_10) | 29 | Panel (17 metabolites) |
|  | Diaz-Arrastia, R[^11^](#_ENREF_11) | 206 | GFAP |
|  | Diaz-Arrastia, R[^11^](#_ENREF_11) | 206 | Ubiquitin |
|  | Diaz-Arrastia, R[^11^](#_ENREF_11) | 206 | Panel (GFAP, ubiquitin) |
|  | Fiandaca, MS[^12^](#_ENREF_12) | 10 | Panel (10 metabolites) |
|  | Kiechle, K[^13^](#_ENREF_13) | 46 | S100B |
|  | Kilianski^[14](#_ENREF_14)^ | 18 | CKBB |
|  | Meier, TB[^15^](#_ENREF_15) | 32 | GFAP |
|  | Meier, TB[^15^](#_ENREF_15) | 64 | S100B |
|  | Meier, TB[^15^](#_ENREF_15) | 32 | UCH-L1 |
|  | Meier, TB[^15^](#_ENREF_15) | 32 | Panel (UCH-L1, S-100B) |
|  | Shahim, P[^16^](#_ENREF_16) | 28 | A-tau |
|  | Shahim, P[^16^](#_ENREF_16) | 28 | C-tau |
|  | Shan, R[^17^](#_ENREF_17) | 55 | copeptin |
|  | Shan, R[^17^](#_ENREF_17) | 55 | LGALS3 |
|  | Shan, R[^17^](#_ENREF_17) | 55 | MMP-9 |
|  | Shan, R[^17^](#_ENREF_17) | 55 | OCLN |
|  | Shan, R[^17^](#_ENREF_17) | 55 | Panel (copeptin, LGALS3, MMP-9) |
|  | Siman, R[^18^](#_ENREF_18) | 28 | SNTF |
|  | Siman, R[^18^](#_ENREF_18) | 55 | Tau-protein |
| **Mild TBI – need for CT Scan** | |  |  |
|  | Asadollahi, S[^19^](#_ENREF_19) | 316 | S100B |
|  | Babcock, L[^20^](#_ENREF_20) | 218 | S100B |
|  | Bazarian, JJ[^21^](#_ENREF_21) | 1947 | Panel (UCH-L1, GFAP) |
|  | Bechtel, K[^22^](#_ENREF_22) | 152 | S100B |
|  | Berger, RP[^23^](#_ENREF_23) | 20 | D-dimer |
|  | Biberthaler, P.[^24^](#_ENREF_24) | 1,309 | S–100B |
|  | Bogoslovsky, T[^25^](#_ENREF_25) | 46 | A-beta-42 |
|  | Bogoslovsky, T[^25^](#_ENREF_25) | 46 | GFAP |
|  | Bogoslovsky, T[^25^](#_ENREF_25) | 46 | Tau |
|  | Bouvier^[26](#_ENREF_26)^ | 446 | S100B |
|  | Bulut, M[^27^](#_ENREF_27) | 60 | tau-proteins |
|  | Castellani, C[^28^](#_ENREF_28) | 109 | S100B |
|  | Diaz-Arrastia R[^11^](#_ENREF_11) | 206 | GFAP |
|  | Diaz-Arrastia R[^11^](#_ENREF_11) | 206 | Ubiquitin |
|  | Egea-Guerrero, JJ[^29^](#_ENREF_29) | 143 | S100B |
|  | Egea-Guerrero, JJ[^30^](#_ENREF_30) | 260 | S100B |
|  | Ernstbrunner, L[^31^](#_ENREF_31) | 382 | S100B |
|  | Gardner, RC[^32^](#_ENREF_32) | 154 | GFAP |
|  | Gardner, RC[^32^](#_ENREF_32) | 154 | P-tau |
|  | Gardner, RC[^32^](#_ENREF_32) | 154 | T-tau |
|  | Gardner, RC[^32^](#_ENREF_32) | 154 | P-tau/ T-tau ratio |
|  | Gatson, JW[^33^](#_ENREF_33) | 68 | NF-H |
|  | Herrmann, M[^34^](#_ENREF_34) | 138 | S100B |
|  | Herrmann, M[^35^](#_ENREF_35) | 132 | NSE |
|  | Herrmann, M[^35^](#_ENREF_35) | 198 | S100B |
|  | Herrmann, M[^36^](#_ENREF_36) | 69 | NSE |
|  | Herrmann, M[^36^](#_ENREF_36) | 69 | S-100B |
|  | Honda, M[^37^](#_ENREF_37) | 34 | GFAP |
|  | Honda, M[^37^](#_ENREF_37) | 34 | NSE |
|  | Honda, M[^37^](#_ENREF_37) | 34 | S100B |
|  | Ingebrigsten, T[^38^](#_ENREF_38) | 50 | S-100 |
|  | Ingebrigtsen, T[^39^](#_ENREF_39) | 182 | S100 |
|  | Kavalci, C[^40^](#_ENREF_40) | 88 | tau-proteins |
|  | Korley, FK[^41^](#_ENREF_41) | 159 | BDNF |
|  | Lagerstedt, L[^42^](#_ENREF_42) | 133 | IL-10 |
|  | Lagerstedt, L[^42^](#_ENREF_42) | 133 | S100B |
|  | Lagerstedt, L[^43^](#_ENREF_43) | 172 | H-FABP |
|  | Lagerstedt, L[^43^](#_ENREF_43) | 516 | S100B |
|  | Manzano, S[^44^](#_ENREF_44) | 201 | S100B |
|  | Martinez-Morillo, E[^45^](#_ENREF_45) | 52 | NFM |
|  | McMahon, PJ[^9^](#_ENREF_9) | 220 | GFAP-BDP |
|  | Metting, Z.[^46^](#_ENREF_46) | 94 | GFAP |
|  | Metting, Z.[^46^](#_ENREF_46) | 94 | S100B |
|  | Mondello, S[^47^](#_ENREF_47) | 45 | GFAP |
|  | Mondello, S[^47^](#_ENREF_47) | 45 | UCH-L1 |
|  | Morochovic, R[^48^](#_ENREF_48) | 102 | S100B |
|  | Müller, B[^49^](#_ENREF_49) | 223 | S-100B |
|  | Muller, K[^50^](#_ENREF_50) | 226 | S100B |
|  | Mussack, T[^51^](#_ENREF_51) | 139 | NSE |
|  | Mussack, T[^51^](#_ENREF_51) | 139 | S100B |
|  | Naeimi, Z[^52^](#_ENREF_52) | 29 | S–100B |
|  | Okonkwo, DO[^53^](#_ENREF_53) | 215 | GFAP-BDP |
|  | Papa, L[^54^](#_ENREF_54) | 108 | GFAP-BDP |
|  | Papa, L[^55^](#_ENREF_55) | 96 | Ubiquitin |
|  | Papa, L[^56^](#_ENREF_56) | 397 | GFAP |
|  | Papa, L[^57^](#_ENREF_57) | 375 | GFAP |
|  | Papa, L[^58^](#_ENREF_58) | 132 | GFAP |
|  | Papa, L[^58^](#_ENREF_58) | 132 | S100B |
|  | Papa, L[^59^](#_ENREF_59) | 1827 | GFAP |
|  | Papa, L[^59^](#_ENREF_59) | 1827 | UCH-L1 |
|  | Peacock, WF[^60^](#_ENREF_60) | 306 | MT3 |
|  | Peacock, WF[^60^](#_ENREF_60) | 494 | NRGN |
|  | Peacock, WF[^60^](#_ENREF_60) | 495 | NSE |
|  | Peacock, WF[^60^](#_ENREF_60) | 299 | Panel (NRGN, NSE, MT3) |
|  | Poli de Figueiredo, LF[^61^](#_ENREF_61) | 50 | S100B |
|  | Posti, JP[^62^](#_ENREF_62) | 1040 | GFAP |
|  | Posti, JP[^62^](#_ENREF_62) | 789 | UCH-L1 |
|  | Romner, B[^63^](#_ENREF_63) | 278 | S-100 |
|  | Rubenstein, R[^64^](#_ENREF_64) | 196 | P-tau |
|  | Rubenstein, R[^64^](#_ENREF_64) | 196 | T-tau |
|  | Rubenstein, R[^64^](#_ENREF_64) | 196 | P-tau/T-tao ratio |
|  | Sharma, R[^65^](#_ENREF_65) | 110 | panel(MMP-2, CRP, CKBB) |
|  | Sharma, R[^65^](#_ENREF_65) | 92 | CKBB |
|  | Sharma, R[^65^](#_ENREF_65) | 92 | CRP |
|  | Sharma, R[^65^](#_ENREF_65) | 92 | GM-CSF |
|  | Sharma, R[^65^](#_ENREF_65) | 92 | hFABP |
|  | Sharma, R[^65^](#_ENREF_65) | 92 | MDA-LDL |
|  | Sharma, R[^65^](#_ENREF_65) | 92 | MMP-2 |
|  | Shaw, GJ[^66^](#_ENREF_66) | 28 | tau-proteins |
|  | Sugimoto, K[^67^](#_ENREF_67) | 73 | D-dimer |
|  | Thaler, HW[^68^](#_ENREF_68) | 782 | S100B |
|  | Welch, RD[^69^](#_ENREF_69) | 251 | S100B |
|  | Welch, RD[^69^](#_ENREF_69) | 251 | UCH-L1 |
|  | Wolf, H[^70^](#_ENREF_70) | 107 | NSE |
|  | Wolf, H[^70^](#_ENREF_70) | 107 | S100B |
|  | Zongo, D[^71^](#_ENREF_71) | 1560 | S100B |
| **Mild TBI – Delayed Recovery** | |  |  |
|  | Babcock, L[^72^](#_ENREF_72) | 76 | S100B |
|  | Bazarian, JJ[^73^](#_ENREF_73) | 35 | S100B |
|  | Bazarian, JJ[^73^](#_ENREF_73) | 35 | tau-protein C |
|  | Bechtel, K[^22^](#_ENREF_22) | 152 | S100B |
|  | Bouvier, D[^26^](#_ENREF_26) | 446 | S100B |
|  | Bulut, M[^27^](#_ENREF_27) | 60 | tau-proteins |
|  | Chen, KY[^74^](#_ENREF_74) | 63 | BMX |
|  | de Boussard, CN[^75^](#_ENREF_75) | 97 | S100B |
|  | Herrmann, M[^36^](#_ENREF_36) | 69 | NSE |
|  | Herrmann, M[^36^](#_ENREF_36) | 138 | S-100B |
|  | Korley, FK[^41^](#_ENREF_41) | 188 | UCH-L1 |
|  | Korley, FK[^41^](#_ENREF_41) | 299 | BDNF |
|  | Korley, FK[^41^](#_ENREF_41) | 188 | GFAP |
|  | Li, M[^76^](#_ENREF_76) | 40 | regulatory T cells |
|  | Ma, M[^77^](#_ENREF_77) | 50 | tau-protein C |
|  | Mannix, R[^78^](#_ENREF_78) | 13 | GFAP |
|  | Metting, Z[^46^](#_ENREF_46) | 94 | GFAP |
|  | Papa, L[^54^](#_ENREF_54) | 108 | GFAP-BDP |
|  | Papa, L[^59^](#_ENREF_59) | 1835 | GFAP |
|  | Papa, L[^59^](#_ENREF_59) | 1835 | UCH-L1 |
|  | Rothoerl, RD[^79^](#_ENREF_79) | 44 | S100B |
|  | Rubenstein, R[^64^](#_ENREF_64) | 134 | P-tau |
|  | Rubenstein, R[^64^](#_ENREF_64) | 134 | T-tau |
|  | Rubenstein, R[^64^](#_ENREF_64) | 134 | P-tau/T-tao ratio |
|  | Ryb, GE[^80^](#_ENREF_80) | 720 | S100B |
|  | Savola, O[^81^](#_ENREF_81) | 516 | S100B |
|  | Shahim, P[^16^](#_ENREF_16) | 56 | tau protein-A |
|  | Shahim, P[^16^](#_ENREF_16) | 56 | tau protein-C |
|  | Shahim, P[^82^](#_ENREF_82) | 35 | NFL |
|  | Siman, R[^83^](#_ENREF_83) | 17 | SNTF |
|  | Siman, R[^18^](#_ENREF_18) | 56 | SNTF |
|  | Stranjalis, G[^84^](#_ENREF_84) | 100 | S100B |
|  | Su, S[^85^](#_ENREF_85) | 846 | CRP |
|  | Topolovec-Vranic, J[^86^](#_ENREF_86) | 140 | NSE |
|  | Topolovec-Vranic, J[^86^](#_ENREF_86) | 140 | S100B |
|  | Wang, HC[^87^](#_ENREF_87) | 68 | sVCAM-1 |
|  | Wilkinson, AA[^88^](#_ENREF_88) | 118 | IL-6 |
|  | Wilkinson, AA[^88^](#_ENREF_88) | 118 | IL-8 |
|  | Wilkinson, AA[^88^](#_ENREF_88) | 118 | NSE |
|  | Wilkinson, AA[^88^](#_ENREF_88) | 118 | pNF-H |
|  | Wilkinson, AA[^88^](#_ENREF_88) | 118 | S100B |
|  | Wilkinson, AA[^88^](#_ENREF_88) | 118 | E-selectin |
|  | Wilkinson, AA[^88^](#_ENREF_88) | 118 | ICAM-1 |
|  | Wilkinson, AA[^88^](#_ENREF_88) | 118 | NCAM |
|  | Wilkinson, AA[^88^](#_ENREF_88) | 118 | VCAM-1 |
|  | Xu, Z[^89^](#_ENREF_89) | 118 | Ghrelin |
| **Severe TBI – Adverse Outcome** | |  |  |
|  | Bandyopadhyay, S[^90^](#_ENREF_90) | 86 | NSE |
|  | Barton, DJ[^91^](#_ENREF_91) | 181 | testosterone |
|  | Bjugstad, KB[^92^](#_ENREF_92) | 104 | icORP |
|  | Brophy, GM[^93^](#_ENREF_93) | 54 | UCH-L1 |
|  | Campello, YV[^94^](#_ENREF_94) | 41 | DNA |
|  | Chabok, SY[^95^](#_ENREF_95) | 28 | NSE |
|  | Chabok, SY[^95^](#_ENREF_95) | 56 | S100B |
|  | Chen, H[^96^](#_ENREF_96) | 34 | neuroglobin |
|  | Chen, H[^96^](#_ENREF_96) | 34 | Nogo-A |
|  | Chen, QH[^97^](#_ENREF_97) | 113 | SCUBE1 |
|  | Chiaretti, A[^98^](#_ENREF_98) | 28 | IL-1beta |
|  | Chiaretti, A[^98^](#_ENREF_98) | 28 | IL-6 |
|  | da Rocha, AB[^99^](#_ENREF_99) | 20 | HSP70 |
|  | da Rocha, AB[^100^](#_ENREF_100) | 46 | S100B |
|  | Dash, PK[^101^](#_ENREF_101) | 20 | ceruloplasmin |
|  | Dash, PK[^101^](#_ENREF_101) | 20 | copper |
|  | De Oliveira, CO[^102^](#_ENREF_102) | 44 | VWF |
|  | DeFazio, MV[^103^](#_ENREF_103) | 88 | D-Dimer |
|  | DeFazio, MV[^103^](#_ENREF_103) | 88 | MMP-9 |
|  | DeFazio, MV[^103^](#_ENREF_103) | 44 | S100B |
|  | DeFazio, MV[^103^](#_ENREF_103) | 44 | S100B |
|  | Diaz-Arrastia R[^11^](#_ENREF_11) | 626 | GFAP |
|  | Diaz-Arrastia, R[^11^](#_ENREF_11) | 626 | UCH-L1 |
|  | Di Battista, AP[^104^](#_ENREF_104) | 170 | BDNF |
|  | Di Battista, AP[^104^](#_ENREF_104) | 170 | GFAP |
|  | Di Battista, AP[^104^](#_ENREF_104) | 170 | ICAM-5 |
|  | Di Battista, AP[^104^](#_ENREF_104) | 170 | MCP-1 |
|  | Di Battista, AP[^104^](#_ENREF_104) | 170 | NSE |
|  | Di Battista, AP[^104^](#_ENREF_104) | 170 | PRDX-6 |
|  | Di Battista, AP[^104^](#_ENREF_104) | 170 | S100B |
|  | Dong, XQ[^105^](#_ENREF_105) | 94 | Copeptin |
|  | Dong, XQ[^106^](#_ENREF_106) | 130 | Periostin |
|  | Dong, XQ[^107^](#_ENREF_107) | 216 | Thioredoxin |
|  | Egea-Guerrero, JJ[^108^](#_ENREF_108) | 140 | S100B |
|  | Feng, MJ[^109^](#_ENREF_109) | 306 | S100A12 protein |
|  | Foaud, HM[^110^](#_ENREF_110) | 138 | D-Dimer |
|  | Fraser, DD[^111^](#_ENREF_111) | 54 | GFAP |
|  | Gonzalez-Mao, MC[^112^](#_ENREF_112) | 149 | S100B |
|  | Jin, Y[^113^](#_ENREF_113) | 228 | Gelsolin |
|  | Karri, J[^114^](#_ENREF_114) | 25 | d-Dimer |
|  | Lei, J[^115^](#_ENREF_115) | 804 | GFAP |
|  | Lee, JY[^116^](#_ENREF_116) | 45 | GFAP |
|  | Lee, JY[^116^](#_ENREF_116) | 45 | S100B |
|  | Lee, JY[^116^](#_ENREF_116) | 45 | UCH-L1 |
|  | Lee, DH[^117^](#_ENREF_117) | 1266 | d_Dimer |
|  | Lee, DH[^117^](#_ENREF_117) | 1266 | FDP |
|  | Lee, DH[^117^](#_ENREF_117) | 1266 | Fibrinogen |
|  | Lee, DH[^117^](#_ENREF_117) | 1266 | INR |
|  | Lee, DH[^117^](#_ENREF_117) | 1266 | platelets |
|  | Li, N[^118^](#_ENREF_118) | 40 | NSE |
|  | Li, N[^118^](#_ENREF_118) | 40 | S100B |
|  | Liliang, PC[^119^](#_ENREF_119) | 34 | tau-proteins |
|  | Lin, C[^120^](#_ENREF_120) | 126 | Copeptin |
|  | Lin, C [^121^](#_ENREF_121) | 284 | Leptin |
|  | Lorente, L[^122^](#_ENREF_122) | 100 | TIMP-1 |
|  | Lorente, L[^123^](#_ENREF_123) | 100 | Substance P |
|  | Lorente, L[^124^](#_ENREF_124) | 100 | MDA |
|  | Lorente, L[^125^](#_ENREF_125) | 100 | Caspase-Cleaved Cytokeratin-18 |
|  | Lorente, L[^126^](#_ENREF_126) | 100 | TAC |
|  | Macher, H[^127^](#_ENREF_127) | 65 | cf-DNA |
|  | Martinez-Morillo, E[^45^](#_ENREF_45) | 12 | NFM |
|  | Meric, E[^128^](#_ENREF_128) | 80 | NSE |
|  | Mondello, S[^129^](#_ENREF_129) | 162 | GFAP |
|  | Mondello, S[^129^](#_ENREF_129) | 162 | UCH-L1 |
|  | Mondello, S[^47^](#_ENREF_47) | 45 | GFAP |
|  | Mondello, S[^47^](#_ENREF_47) | 45 | UCH-L1 |
|  | Mortberg, E[^130^](#_ENREF_130) | 88 | tau- proteins |
|  | Murillo-Cabezas, F[^131^](#_ENREF_131) | 696 | S100B |
|  | Mussack, T[^132^](#_ENREF_132) | 20 | IL-8 |
|  | Mussack, T[^132^](#_ENREF_132) | 20 | S100B |
|  | Nylen, K[^133^](#_ENREF_133) | -354 | GFAP |
|  | Nylen, K[^134^](#_ENREF_134) | 59 | S100A1B |
|  | Nylen, K[^134^](#_ENREF_134) | 59 | S100B |
|  | Okonkwo, DO[^53^](#_ENREF_53) | 215 | GFAP-BDP |
|  | Olivecrona, Z[^135^](#_ENREF_135) | 96 | NSE |
|  | Olivecrona, Z[^135^](#_ENREF_135) | 96 | S-100B |
|  | Pan, JW[^136^](#_ENREF_136) | 384 | ficolin-3 |
|  | Pelinka, LE[^137^](#_ENREF_137) | 230 | S100B |
|  | Petzold, A[^138^](#_ENREF_138) | 21 | S100B |
|  | Petzold, A[^138^](#_ENREF_138) | 21 | S100B |
|  | Pleines, UE[^139^](#_ENREF_139) | 13 | ICAM1 |
|  | Raabe, A[^140^](#_ENREF_140) | 44 | S100B |
|  | Raabe, A[^141^](#_ENREF_141) | 84 | S-100B |
|  | Raabe, AR[^142^](#_ENREF_142) | 82 | NSE |
|  | Raabe, AR[^142^](#_ENREF_142) | 82 | S100B |
|  | Raheja, A[^143^](#_ENREF_143) | 258 | GFAP |
|  | Raheja, A[^143^](#_ENREF_143) | 258 | IL-6 |
|  | Rainey, TR[^144^](#_ENREF_144) | 100 | S100B |
|  | Randall, J[^145^](#_ENREF_145) | 75 | tau-proteins |
|  | Rodriguez-Rodriguez, A[^146^](#_ENREF_146) | 55 | S100B |
|  | Rodriguez-Rodriguez, A[^147^](#_ENREF_147) | 198 | NSE |
|  | Rodriguez-Rodriguez, A[^147^](#_ENREF_147) | 990 | S100B |
|  | Sadaka, F[^148^](#_ENREF_148) | 416 | RDW |
|  | Schneider Soares, FS [^149^](#_ENREF_149) | 426 | IL-10 |
|  | Shahim, P[^150^](#_ENREF_150) | 70 | NFL |
|  | Shallwani, H[^151^](#_ENREF_151) | 216 | base deficit |
|  | Shaw, GJ[^152^](#_ENREF_152) | 28 | tau-proteins |
|  | Shen, L[^153^](#_ENREF_153) | 86 | Adiponectin |
|  | Shen, YF[^154^](#_ENREF_154) | 300 | galectin-3 |
|  | Simon, D[^155^](#_ENREF_155) | 69 | ferritin |
|  | Simon, D[^156^](#_ENREF_156) | 80 | MMP-9 |
|  | Spinella, PC[^157^](#_ENREF_157) | 27 | S100B |
|  | Takala, RS[^158^](#_ENREF_158) | 130 | GFAP |
|  | Takala, RS[^158^](#_ENREF_158) | 130 | UCH-L1 |
|  | Vos, PE[^159^](#_ENREF_159) | 170 | GFAP |
|  | Vos, PE[^159^](#_ENREF_159) | 170 | NSE |
|  | Vos, PE[^159^](#_ENREF_159) | 170 | S100B |
|  | Vos, PE[^160^](#_ENREF_160) | 158 | GFAP |
|  | Vos, PE[^160^](#_ENREF_160) | 158 | S100B |
|  | Walder, BW[^161^](#_ENREF_161) | 49 | H-FABP |
|  | Wang, HC[^162^](#_ENREF_162) | 88 | GSH |
|  | Wang, J[^163^](#_ENREF_163) | 56 | tau-proteins |
|  | Wang, JL[^164^](#_ENREF_164) | 402 | thrombospondin-1 |
|  | Wang, KY[^165^](#_ENREF_165) | 212 | HMGB1 |
|  | Woertgen, C[^166^](#_ENREF_166) | 44 | S100B |
|  | Woertgen, C[^167^](#_ENREF_167) | 51 | S-100B |
|  | Woiciechowsky, C[^168^](#_ENREF_168) | 51 | IL-6 |
|  | Wu, GQ[^169^](#_ENREF_169) | 200 | nesfatin-1 |
|  | Xu, JF[^170^](#_ENREF_170) | 94 | Gelsolin |
|  | Yamazaki, Y[^171^](#_ENREF_171) | 25 | MBP |
|  | Yamazaki, Y[^171^](#_ENREF_171) | 25 | NSE |
|  | Yang, DB[^172^](#_ENREF_172) | 100 | Copeptin |
|  | Yang, DB[^173^](#_ENREF_173) | 216 | MIF |
|  | Yu, LY[^174^](#_ENREF_174) | 78 | suPAR |
|  | Yu, W[^175^](#_ENREF_175) | 244 | MBL |
|  | Zhang, B[^176^](#_ENREF_176) | 188 | Cholinesterase |
|  | Zhang, B[^177^](#_ENREF_177) | 122 | RDW |
|  | Zhang, ZY[^178^](#_ENREF_178) | 102 | Copeptin |
|  | Zhang, ZY[^178^](#_ENREF_178) | 102 | GFAP |
|  | Zhang, ZY[^178^](#_ENREF_178) | 102 | MBP |
|  | Zhang, ZY[^178^](#_ENREF_178) | 102 | NF-H |
|  | Zhang, ZY[^178^](#_ENREF_178) | 102 | NSE |
|  | Zhang, ZY[^178^](#_ENREF_178) | 102 | S100B |
|  | Zhang, ZY[^178^](#_ENREF_178) | 102 | tau-proteins |
|  | Zhang, ZY[^178^](#_ENREF_178) | 102 | UCH-L1 |
|  | Zhao, YY[^179^](#_ENREF_179) | 216 | Tenascin-C |
|  | Zurek, J[^180^](#_ENREF_180) | 98 | NF-H |

**Table S2: Operating Characteristics of Theoretical Test**

| **Cut Point**  **(units)** | **Sensitivity** | **Specificity** | **J-statistic** |
| --- | --- | --- | --- |
| 0 | 1.0 | 0 | 0 |
| 1 | 1.0 | 0.1 | 0.1 |
| 2 | 0.8 | 0.4 | 0.2 |
| 3 | 0.8 | 0.6 | **0.4** |
| 4 | 0.6 | 0.7 | 0.3 |
| 5 | 0.4 | 0.9 | 0.3 |
| 6 | 0 | 1.0 | 0 |

**Legends for Supplementary Figures**

Fig S1: Illustration of theoretical test results for 5 patients positive for a given disease and 10 negative patients for that disease. Each patient is shown as a dot in the appropriate column. A set of cut points are shown by the horizontal dotted lines. The cut point is the value above which the test is considered positive.

Fig S2: A 2X2 contingency table to calculate the operating characteristics of a test. TP = number of true positive tests; TN = true negative; FP = false positive; FN = false negative. The number of true positive tests, divided by the number with the disease (TP + FN) equals the test’s sensitivity. The number of true negatives, divided by all without disease (TN + FP) equals the test’s specificity.

Fig S3: For each of the cut points in our theoretical test, the calculated sensitivities are plotted against 1 minus the specificities. Joining the points yields the receiver operating curve (ROC). The area under the curve (AUC) can be calculated to measure the test’s ability to discriminate between subjects with and without disease.

Fig S4: Comparing two ROC curves with identical AUC’s and identical J-statistics. The difference between the two (which is more “accurate”) depends on which errors are more costly. Curve A (solid line) has fewer false positive results, whereas Curve B (dashed line) has fewer false negative results. In the case of mild TBI and indications for CT scan, missed hematomas (false negative) are much more costly than false positives (unnecessary CT scans).

**Fig. S1**


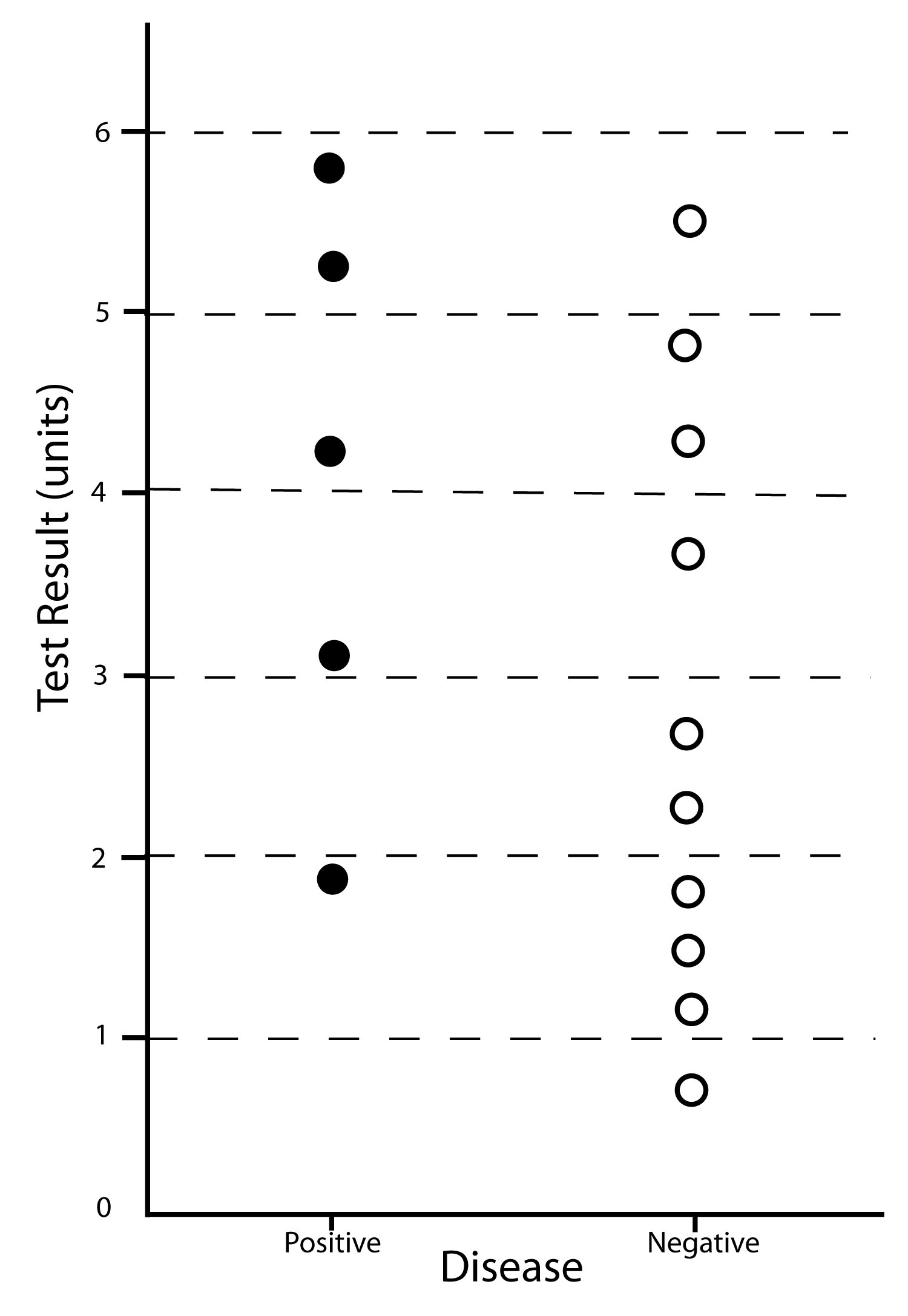


**Fig. S2**


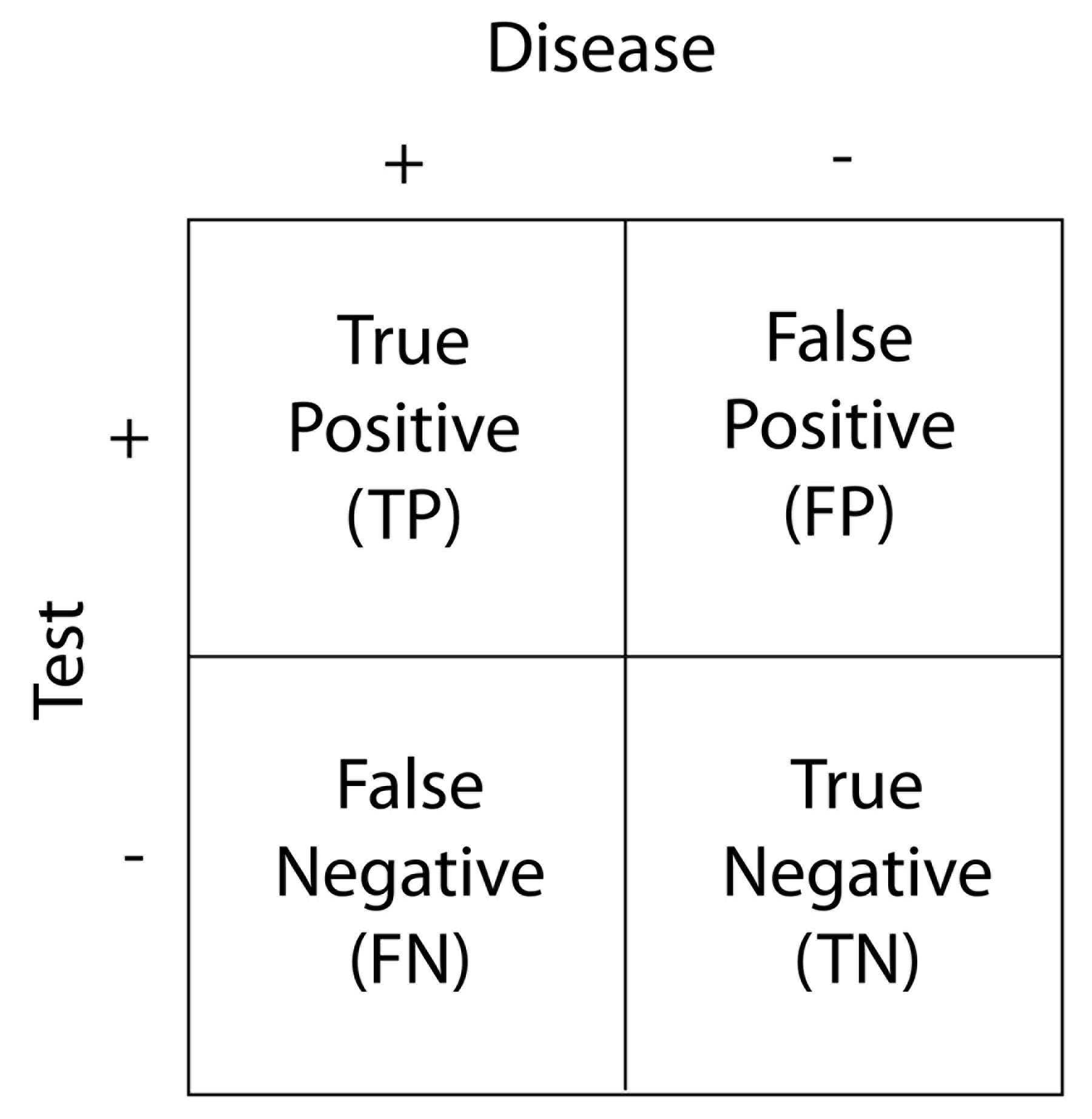


**Fig S3**


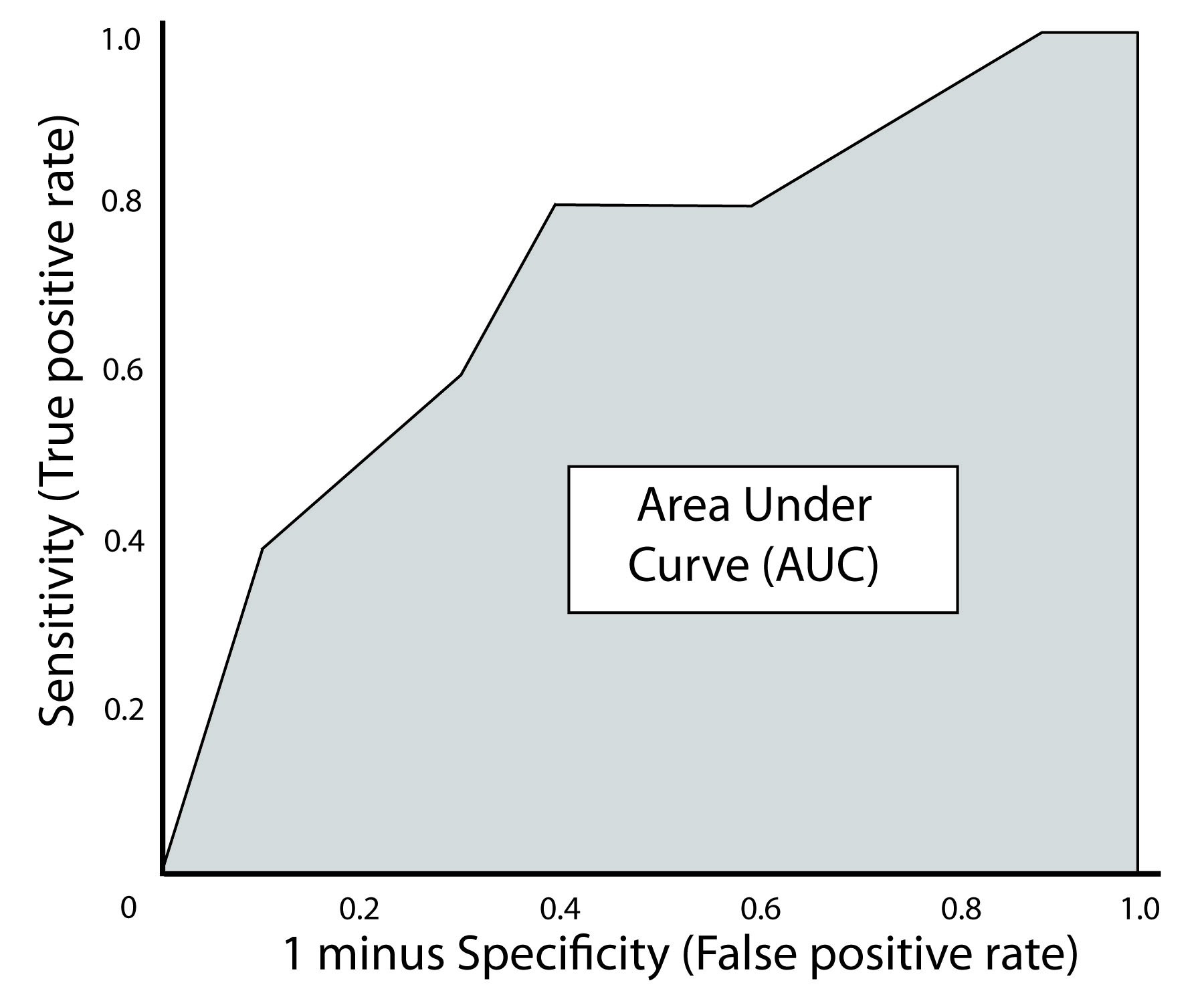


**Fig S4**


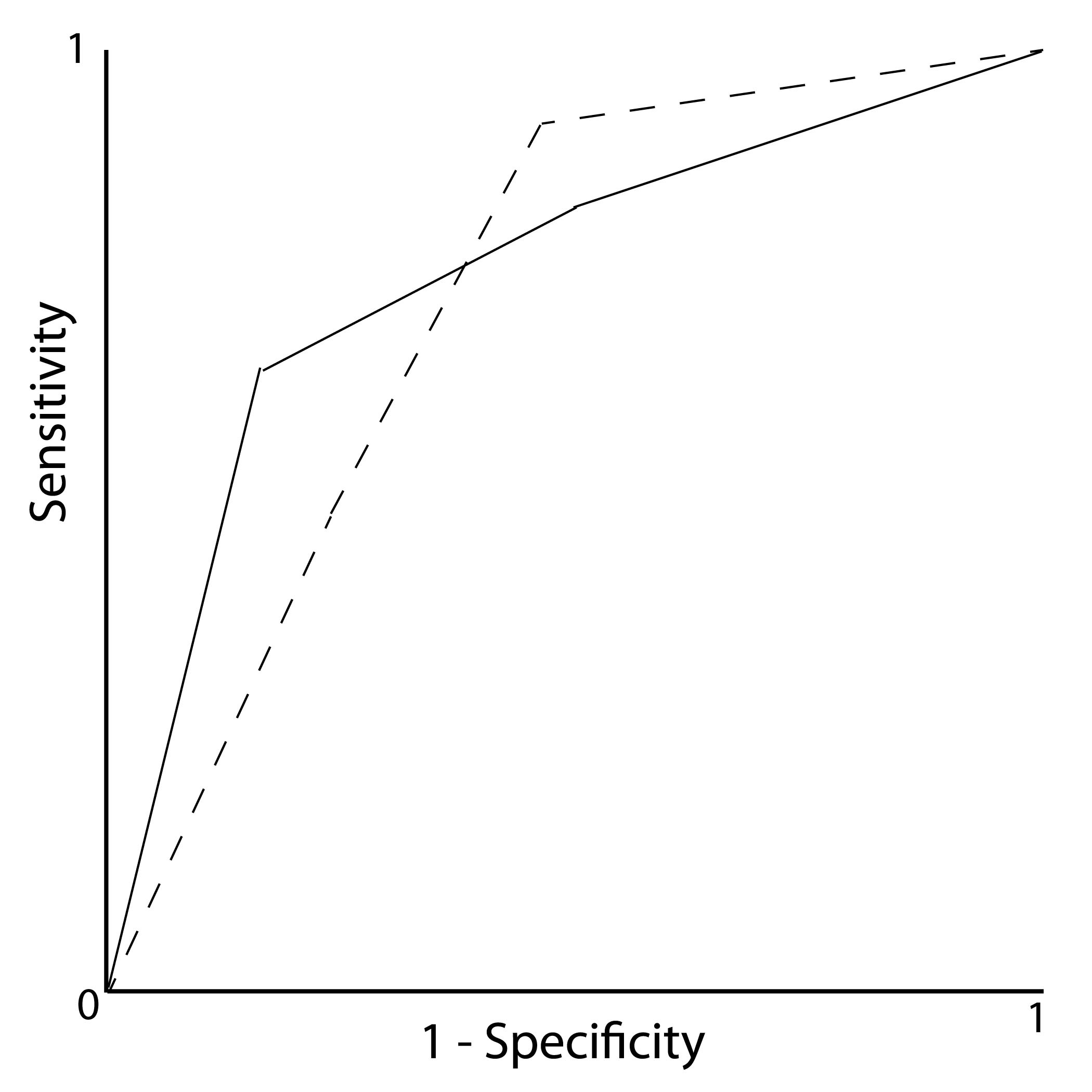


**References**

1. Sox, H.C., Blatt, M.A., Higgins, M.C. and Marton, K.I. (2007). Medical Decision Making. 2 ed. American College of Physicians: Philadelphia.

2. Linden, A. (2006). Measuring diagnostic and predictive accuracy in disease management: an introduction to receiver operating characteristic (ROC) analysis. Journal of evaluation in clinical practice 12, 132-139.

3. Metz, C.E. (1978). Basic principles of ROC analysis. Seminars in nuclear medicine 8, 283-298.

4. Youden, W.J. (1950). Index for rating diagnostic tests. Cancer 3, 32-35.

5. Miller, J.D., Tocher, J.L. and Jones, P.A. (1988). Extradural haematoma--earlier detection, better results. Brain injury 2, 83-86.

6. Stein, S.C., Burnett, M.G. and Glick, H.A. (2006). Indications for CT scanning in mild traumatic brain injury: A cost-effectiveness study. The Journal of trauma 61, 558-566.

7. Stiell, I.G., Wells, G.A., Vandemheen, K., Clement, C., Lesiuk, H., Laupacis, A., McKnight, R.D., Verbeek, R., Brison, R., Cass, D., Eisenhauer, M.E., Greenberg, G. and Worthington, J. (2001). The Canadian CT Head Rule for patients with minor head injury. Lancet 357, 1391-1396.

8. Stein, S.C., Fabbri, A., Servadei, F. and Glick, H.A. (2009). A critical comparison of clinical decision instruments for computed tomographic scanning in mild closed traumatic brain injury in adolescents and adults. Annals of emergency medicine 53, 180-188.

9. McMahon, P.J., Panczykowski, D.M., Yue, J.K., Puccio, A.M., Inoue, T., Sorani, M.D., Lingsma, H.F., Maas, A.I., Valadka, A.B., Yuh, E.L., Mukherjee, P., Manley, G.T., Okonkwo, D.O., Casey, S.S., Cheong, M., Cooper, S.R., Dams-O'Connor, K., Gordon, W.A., Hricik, A.J., Lawless, K., Menon, D., Schnyer, D.M. and Vassar, M.J. (2015). Measurement of the glial fibrillary acidic protein and its breakdown products GFAP-BDP biomarker for the detection of traumatic brain injury compared to computed tomography and magnetic resonance imaging. Journal of neurotrauma 32, 527-533.

10. Daley, M., Dekaban, G., Bartha, R., Brown, A., Stewart, T.C., Doherty, T., Fischer, L., Holmes, J., Menon, R.S., Rupar, C.A., Shoemaker, J.K. and Fraser, D.D. (2016). Metabolomics profiling of concussion in adolescent male hockey players: a novel diagnostic method. Metabolomics 12, 185.

11. Diaz-Arrastia, R., Wang, K.K., Papa, L., Sorani, M.D., Yue, J.K., Puccio, A.M., McMahon, P.J., Inoue, T., Yuh, E.L., Lingsma, H.F., Maas, A.I., Valadka, A.B., Okonkwo, D.O. and Manley, G.T. (2014). Acute biomarkers of traumatic brain injury: relationship between plasma levels of ubiquitin C-terminal hydrolase-L1 and glial fibrillary acidic protein. Journal of neurotrauma 31, 19-25.

12. Fiandaca, M.S., Mapstone, M., Mahmoodi, A., Gross, T., Macciardi, F., Cheema, A.K., Merchant-Borna, K., Bazarian, J. and Federoff, H.J. (2018). Plasma metabolomic biomarkers accurately classify acute mild traumatic brain injury from controls. PloS one 13, e0195318.

13. Kiechle, K., Bazarian, J.J., Merchant-Borna, K., Stoecklein, V., Rozen, E., Blyth, B., Huang, J.H., Dayawansa, S., Kanz, K. and Biberthaler, P. (2014). Subject-specific increases in serum S-100B distinguish sports-related concussion from sports-related exertion. PloS one 9, e84977.

14. Kilianski, J., Peeters, S., Debad, J., Mohmed, J., Wolf, S.E., Minei, J.P., Diaz-Arrastia, R. and Gatson, J.W. (2017). Plasma creatine kinase B correlates with injury severity and symptoms in professional boxers. Journal of clinical neuroscience : official journal of the Neurosurgical Society of Australasia 45, 100-104.

15. Meier, T.B., Nelson, L.D., Huber, D.L., Bazarian, J.J., Hayes, R.L. and McCrea, M.A. (2017). Prospective Assessment of Acute Blood Markers of Brain Injury in Sport-Related Concussion. Journal of neurotrauma 34, 3134-3142.

16. Shahim, P., Linemann, T., Inekci, D., Karsdal, M.A., Blennow, K., Tegner, Y., Zetterberg, H. and Henriksen, K. (2015). Serum tau fragments predict return to play in concussed professional ice hockey players. Journal of neurotrauma.

17. Shan, R., Szmydynger-Chodobska, J., Warren, O.U., Mohammad, F., Zink, B.J. and Chodobski, A. (2016). A New Panel of Blood Biomarkers for the Diagnosis of Mild Traumatic Brain Injury/Concussion in Adults. Journal of neurotrauma 33, 49-57.

18. Siman, R., Shahim, P., Tegner, Y., Blennow, K., Zetterberg, H. and Smith, D.H. (2015). Serum SNTF Increases in Concussed Professional Ice Hockey Players and Relates to the Severity of Postconcussion Symptoms. Journal of neurotrauma 32, 1294-1300.

19. Asadollahi, S., Heidari, K., Taghizadeh, M., Seidabadi, A.M., Jamshidian, M., Vafaee, A., Manoochehri, M., Shojaee, A.H. and Hatamabadi, H.R. (2015). Reducing head computed tomography after mild traumatic brain injury: Screening value of clinical findings and S100B protein levels. Brain injury 30, 172-178.

20. Babcock, L., Byczkowski, T., Mookerjee, S. and Bazarian, J.J. (2012). Ability of S100B to predict severity and cranial CT results in children with TBI. Brain injury 26, 1372-1380.

21. Bazarian, J.J., Biberthaler, P., Welch, R.D., Lewis, L.M., Barzo, P., Bogner-Flatz, V., Gunnar Brolinson, P., Buki, A., Chen, J.Y., Christenson, R.H., Hack, D., Huff, J.S., Johar, S., Jordan, J.D., Leidel, B.A., Lindner, T., Ludington, E., Okonkwo, D.O., Ornato, J., Peacock, W.F., Schmidt, K., Tyndall, J.A., Vossough, A. and Jagoda, A.S. (2018). Serum GFAP and UCH-L1 for prediction of absence of intracranial injuries on head CT (ALERT-TBI): a multicentre observational study. The Lancet. Neurology.

22. Bechtel, K., Frasure, S., Marshall, C., Dziura, J. and Simpson, C. (2009). Relationship of serum S100B levels and intracranial injury in children with closed head trauma. Pediatrics 124, e697-704.

23. Berger, R.P., Fromkin, J., Rubin, P., Snyder, J., Richichi, R. and Kochanek, P. (2015). Serum D-Dimer Concentrations Are Increased after Pediatric Traumatic Brain Injury. The Journal of pediatrics 166, 383-388.

24. Biberthaler, P., Linsenmeier, U., Pfeifer, K.J., Kroetz, M., Mussack, T., Kanz, K.G., Hoecherl, E.F., Jonas, F., Marzi, I., Leucht, P., Jochum, M. and Mutschler, W. (2006). Serum S-100B concentration provides additional information fot the indication of computed tomography in patients after minor head injury: a prospective multicenter study. Shock 25, 446-453.

25. Bogoslovsky, T., Wilson, D., Chen, Y., Hanlon, D., Gill, J., Jeromin, A., Song, L., Moore, C., Gong, Y., Kenney, K. and Diaz-Arrastia, R. (2017). Increases of Plasma Levels of Glial Fibrillary Acidic Protein, Tau, and Amyloid beta up to 90 Days after Traumatic Brain Injury. Journal of neurotrauma 34, 66-73.

26. Bouvier, D., Fournier, M., Dauphin, J.B., Amat, F., Ughetto, S., Labbe, A. and Sapin, V. (2012). Serum S100B determination in the management of pediatric mild traumatic brain injury. Clinical chemistry 58, 1116-1122.

27. Bulut, M., Koksal, O., Dogan, S., Bolca, N., Ozguc, H., Korfali, E., Ilcol, Y.O. and Parklak, M. (2006). Tau protein as a serum marker of brain damage in mild traumatic brain injury: preliminary results. Advances in therapy 23, 12-22.

28. Castellani, C., Bimbashi, P., Ruttenstock, E., Sacherer, P., Stojakovic, T. and Weinberg, A.M. (2009). Neuroprotein s-100B -- a useful parameter in paediatric patients with mild traumatic brain injury? Acta Paediatr 98, 1607-1612.

29. Egea-Guerrero, J.J., Revuelto-Rey, J., Murillo-Cabezas, F., Munoz-Sanchez, M.A., Vilches-Arenas, A., Sanchez-Linares, P., Dominguez-Roldan, J.M. and Leon-Carrion, J. (2012). Accuracy of the S100beta protein as a marker of brain damage in traumatic brain injury. Brain injury 26, 76-82.

30. Egea-Guerrero, J.J., Rodriguez-Rodriguez, A., Quintana-Diaz, M., Freire-Aragon, M.D., Raya-Collados, D., Hernandez-Garcia, C., Ortiz-Manzano, A., Vilches-Arenas, A., Diez-Naz, A., Guerrero, J.M. and Murillo-Cabezas, F. (2018). Validation of S100B use in a cohort of Spanish patients with mild traumatic brain injury: a multicentre study. Brain injury 32, 459-463.

31. Ernstbrunner, L., Korn, G., Ernstbrunner, E., Auffarth, A., Tauber, M., Resch, H. and Moroder, P. (2015). S100B serum protein cannot predict secondary intracranial haemorrhage after mild head injury in patients with low-dose acetylsalicylic acid prophylaxis. Brain injury 30, 43-47.

32. Gardner, R.C., Rubenstein, R., Wang, K.K.W., Korley, F.K., Yue, J.K., Yuh, E.L., Mukherjee, P., Valadka, A., Okonkwo, D.O., Diaz-Arrastia, R. and Manley, G. (2018). Age-Related Differences in Diagnostic Accuracy of Plasma GFAP and Tau For Identifying Acute Intracranial Trauma on CT: A TRACK-TBI Study. Journal of neurotrauma.

33. Gatson, J.W., Barillas, J., Hynan, L.S., Diaz-Arrastia, R., Wolf, S.E. and Minei, J.P. (2014). Detection of neurofilament-H in serum as a diagnostic tool to predict injury severity in patients who have suffered mild traumatic brain injury. Journal of neurosurgery 121, 1232-1238.

34. Herrmann, M., Curio, N., Jost, S., Wunderlich, M.T., Synowitz, H. and Wallesch, C.W. (1999). Protein S-100B and neuron specific enolase as early neurobiochemical markers of the severity of traumatic brain injury. Restorative neurology and neuroscience 14, 109-114.

35. Herrmann, M., Jost, S., Kutz, S., Ebert, A.D., Kratz, T., Wunderlich, M.T. and Synowitz, H. (2000). Temporal profile of release of neurobiochemical markers of brain damage after traumatic brain injury is associated with intracranial pathology as demonstrated in cranial computerized tomography. Journal of neurotrauma 17, 113-122.

36. Herrmann, M., Curio, N., Jost, S., Grubich, C., Ebert, A.D., Fork, M.L. and Synowitz, H. (2001). Release of biochemical markers of damage to neuronal and glial brain tissue is associated with short and long term neuropsychological outcome after traumatic brain injury. Journal of neurology, neurosurgery, and psychiatry 70, 95-100.

37. Honda, M., Tsuruta, R., Kaneko, T., Kasaoka, S., Yagi, T., Todani, M., Fujita, M., Izumi, T. and Maekawa, T. (2010). Serum glial fibrillary acidic protein is a highly specific biomarker for traumatic brain injury in humans compared with S-100B and neuron-specific enolase. The Journal of trauma 69, 104-109.

38. Ingebrigtsen, T., Waterloo, K., Jacobsen, E.A., Langbakk, B. and Romner, B. (1999). Traumatic brain damage in minor head injury: relation of serum S-100 protein measurements to magnetic resonance imaging and neurobehavioral outcome. Neurosurgery 45, 468-475; discussion 475-466.

39. Ingebrigtsen, T., Romner, B., Marup-Jensen, S., Dons, M., Lundqvist, C., Bellner, J., Alling, C. and Borgesen, S.E. (2000). The clinical value of serum S-100 protein measurements in minor head injury: a Scandinavian multicentre study. Brain injury 14, 1047-1055.

40. Kavalci, C., Pekdemir, M., Durukan, P., Ilhan, N., Yildiz, M., Serhatlioglu, S. and Seckin, D. (2007). The value of serum tau protein for the diagnosis of intracranial injury in minor head trauma. The American journal of emergency medicine 25, 391-395.

41. Korley, F.K., Diaz-Arrastia, R., Wu, A.H., Yue, J.K., Manley, G.T., Sair, H.I., Van Eyk, J., Everett, A.D., Okonkwo, D.O., Valadka, A.B., Gordon, W.A., Maas, A.I., Mukherjee, P., Yuh, E.L., Lingsma, H.F., Puccio, A.M. and Schnyer, D.M. (2016). Circulating Brain-Derived Neurotrophic Factor Has Diagnostic and Prognostic Value in Traumatic Brain Injury. Journal of neurotrauma 33, 215-225.

42. Lagerstedt, L., Egea-Guerrero, J.J., Rodriguez-Rodriguez, A., Bustamante, A., Montaner, J., El Rahal, A., Andereggen, E., Rinaldi, L., Sarrafzadeh, A., Schaller, K. and Sanchez, J.C. (2018). Early measurement of interleukin-10 predicts the absence of CT scan lesions in mild traumatic brain injury. PloS one 13, e0193278.

43. Lagerstedt, L., Egea-Guerrero, J.J., Bustamante, A., Montaner, J., Rodriguez-Rodriguez, A., El Rahal, A., Turck, N., Quintana, M., Garcia-Armengol, R., Prica, C.M., Andereggen, E., Rinaldi, L., Sarrafzadeh, A., Schaller, K. and Sanchez, J.C. (2017). H-FABP: A new biomarker to differentiate between CT-positive and CT-negative patients with mild traumatic brain injury. PloS one 12, e0175572.

44. Manzano, S., Holzinger, I.B., Kellenberger, C.J., Lacroix, L., Klima-Lange, D., Hersberger, M., La Scala, G., Altermatt, S. and Staubli, G. (2016). Diagnostic performance of S100B protein serum measurement in detecting intracranial injury in children with mild head trauma. Emergency Medicine Journal 33, 42-46.

45. Martínez-Morillo, E., Childs, C., García, B.P., Álvarez Menéndez, F.V., Romaschin, A.D., Cervellin, G., Lippi, G. and Diamandis, E.P. (2015). Neurofilament medium polypeptide (NFM) protein concentration is increased in CSF and serum samples from patients with brain injury. Clinical Chemistry and Laboratory Medicine (CCLM) 53.

46. Metting, Z., Wilczak, N., Rodiger, L.A., Schaaf, J.M. and van der Naalt, J. (2012). GFAP and S100B in the acute phase of mild traumatic brain injury. Neurology 78, 1428-1433.

47. Mondello, S., Kobeissy, F., Vestri, A., Hayes, R.L., Kochanek, P.M. and Berger, R.P. (2016). Serum Concentrations of Ubiquitin C-Terminal Hydrolase-L1 and Glial Fibrillary Acidic Protein after Pediatric Traumatic Brain Injury. Scientific Reports 6, 28203.

48. Morochovic, R., Racz, O., Kitka, M., Pingorova, S., Cibur, P., Tomkova, D. and Lenartova, R. (2009). Serum S100B protein in early management of patients after mild traumatic brain injury. European journal of neurology : the official journal of the European Federation of Neurological Societies 16, 1112-1117.

49. Muller, B., Evangelopoulos, D.S., Bias, K., Wildisen, A., Zimmermann, H. and Exadaktylos, A.K. (2011). Can S-100B serum protein help to save cranial CT resources in a peripheral trauma centre? A study and consensus paper. Emergency medicine journal : EMJ 28, 938-940.

50. Muller, K., Townend, W., Biasca, N., Unden, J., Waterloo, K., Romner, B. and Ingebrigtsen, T. (2007). S100B serum level predicts computed tomography findings after minor head injury. The Journal of trauma 62, 1452-1456.

51. Mussack, T., Biberthaler, P., Kanz, K.G., Heckl, U., Gruber, R., Linsenmaier, U., Mutschler, W. and Jochum, M. (2002). Immediate S-100B and neuron-specific enolase plasma measurements for rapid evaluation of primary brain damage in alcohol-intoxicated, minor head-injured patients. Shock 18, 395-400.

52. Naeimi, Z.S., Weinhofer, A., Sarahrudi, K., Heinz, T. and Vecsei, V. (2006). Predictive value of S-100B protein and neuron specific-enolase as markers of traumatic brain damage in clinical use. Brain injury 20, 463-468.

53. Okonkwo, D.O., Yue, J.K., Puccio, A.M., Panczykowski, D.M., Inoue, T., McMahon, P.J., Sorani, M.D., Yuh, E.L., Lingsma, H.F., Maas, A.I., Valadka, A.B. and Manley, G.T. (2013). GFAP-BDP as an acute diagnostic marker in traumatic brain injury: results from the prospective transforming research and clinical knowledge in traumatic brain injury study. Journal of neurotrauma 30, 1490-1497.

54. Papa, L., Lewis, L.M., Falk, J.L., Zhang, Z., Silvestri, S., Giordano, P., Brophy, G.M., Demery, J.A., Dixit, N.K., Ferguson, I., Liu, M.C., Mo, J., Akinyi, L., Schmid, K., Mondello, S., Robertson, C.S., Tortella, F.C., Hayes, R.L. and Wang, K.K. (2012). Elevated levels of serum glial fibrillary acidic protein breakdown products in mild and moderate traumatic brain injury are associated with intracranial lesions and neurosurgical intervention. Annals of emergency medicine 59, 471-483.

55. Papa, L., Lewis, L.M., Silvestri, S., Falk, J.L., Giordano, P., Brophy, G.M., Demery, J.A., Liu, M.C., Mo, J., Akinyi, L., Mondello, S., Schmid, K., Robertson, C.S., Tortella, F.C., Hayes, R.L. and Wang, K.K. (2012). Serum levels of ubiquitin C-terminal hydrolase distinguish mild traumatic brain injury from trauma controls and are elevated in mild and moderate traumatic brain injury patients with intracranial lesions and neurosurgical intervention. The journal of trauma and acute care surgery 72, 1335-1344.

56. Papa, L., Silvestri, S., Brophy, G.M., Giordano, P., Falk, J.L., Braga, C.F., Tan, C.N., Ameli, N.J., Demery, J.A., Dixit, N.K., Mendes, M.E., Hayes, R.L., Wang, K.K. and Robertson, C.S. (2014). GFAP out-performs S100beta in detecting traumatic intracranial lesions on computed tomography in trauma patients with mild traumatic brain injury and those with extracranial lesions. Journal of neurotrauma 31, 1815-1822.

57. Papa, L., Zonfrillo, M.R., Ramirez, J., Silvestri, S., Giordano, P., Braga, C.F., Tan, C.N., Ameli, N.J., Lopez, M., Mittal, M.K. and Macy, M. (2015). Performance of Glial Fibrillary Acidic Protein in Detecting Traumatic Intracranial Lesions on Computed Tomography in Children and Youth With Mild Head Trauma. Academic Emergency Medicine 22, 1274-1282.

58. Papa, L., Mittal, M.K., Ramirez, J., Ramia, M., Kirby, S., Silvestri, S., Giordano, P., Weber, K., Braga, C.F., Tan, C.N., Ameli, N.J., Lopez, M. and Zonfrillo, M.R. (2016). In Children and Youth with Mild and Moderate Traumatic Brain Injury, Glial Fibrillary Acidic Protein Out-Performs S100β in Detecting Traumatic Intracranial Lesions on Computed Tomography. Journal of neurotrauma 33, 58-64.

59. Papa, L., Brophy, G.M., Welch, R.D., Lewis, L.M., Braga, C.F., Tan, C.N., Ameli, N.J., Lopez, M.A., Haeussler, C.A., Mendez Giordano, D.I., Silvestri, S., Giordano, P., Weber, K.D., Hill-Pryor, C. and Hack, D.C. (2016). Time Course and Diagnostic Accuracy of Glial and Neuronal Blood Biomarkers GFAP and UCH-L1 in a Large Cohort of Trauma Patients With and Without Mild Traumatic Brain Injury. JAMA neurology 73, 551.

60. Peacock, W.F.t., Van Meter, T.E., Mirshahi, N., Ferber, K., Gerwien, R., Rao, V., Sair, H.I., Diaz-Arrastia, R. and Korley, F.K. (2017). Derivation of a Three Biomarker Panel to Improve Diagnosis in Patients with Mild Traumatic Brain Injury. Frontiers in neurology 8, 641.

61. Poli-de-Figueiredo, L.F., Biberthaler, P., Simao Filho, C., Hauser, C., Mutschler, W. and Jochum, M. (2006). Measurement of S-100B for risk classification of victims sustaining minor head injury--first pilot study in Brazil. Clinics (Sao Paulo) 61, 41-46.

62. Posti, J.P., Takala, R.S.K., Runtti, H., Newcombe, V.F., Outtrim, J., Katila, A.J., Frantzén, J., Ala-Seppälä, H., Coles, J.P., Hossain, M.I., Kyllönen, A., Maanpää, H.-R., Tallus, J., Hutchinson, P.J., van Gils, M., Menon, D.K. and Tenovuo, O. (2016). The Levels of Glial Fibrillary Acidic Protein and Ubiquitin C-Terminal Hydrolase-L1 During the First Week After a Traumatic Brain Injury. Neurosurgery 79, 456-464.

63. Romner, B., Ingebrigtsen, T., Kongstad, P. and Borgesen, S.E. (2000). Traumatic brain damage: serum S-100 protein measurements related to neuroradiological findings. Journal of neurotrauma 17, 641-647.

64. Rubenstein, R., Chang, B., Yue, J.K., Chiu, A., Winkler, E.A., Puccio, A.M., Diaz-Arrastia, R., Yuh, E.L., Mukherjee, P., Valadka, A.B., Gordon, W.A., Okonkwo, D.O., Davies, P., Agarwal, S., Lin, F., Sarkis, G., Yadikar, H., Yang, Z., Manley, G.T., Wang, K.K.W., Cooper, S.R., Dams-O'Connor, K., Borrasso, A.J., Inoue, T., Maas, A.I.R., Menon, D.K., Schnyer, D.M. and Vassar, M.J. (2017). Comparing Plasma Phospho Tau, Total Tau, and Phospho Tau-Total Tau Ratio as Acute and Chronic Traumatic Brain Injury Biomarkers. JAMA neurology 74, 1063-1072.

65. Sharma, R., Rosenberg, A., Bennett, E.R., Laskowitz, D.T. and Acheson, S.K. (2017). A blood-based biomarker panel to risk-stratify mild traumatic brain injury. PloS one 12, e0173798.

66. Shaw, G., Yang, C., Ellis, R., Anderson, K., Parker Mickle, J., Scheff, S., Pike, B., Anderson, D.K. and Howland, D.R. (2005). Hyperphosphorylated neurofilament NF-H is a serum biomarker of axonal injury. Biochemical and biophysical research communications 336, 1268-1277.

67. Sugimoto, K., Suehiro, E., Shinoyama, M., Sadahiro, H., Haji, K., Fujiyama, Y., Kawano, R., Nishioka, M. and Suzuki, M. (2017). D-Dimer Elevation as a Blood Biomarker for Detection of Structural Disorder in Mild Traumatic Brain Injury. Journal of neurotrauma 34, 3245-3248.

68. Thaler, H.W., Schmidsfeld, J., Pusch, M., Pienaar, S., Wunderer, J., Pittermann, P., Valenta, R., Gleiss, A., Fialka, C. and Mousavi, M. (2015). Evaluation of S100B in the diagnosis of suspected intracranial hemorrhage after minor head injury in patients who are receiving platelet aggregation inhibitors and in patients 65 years of age and older. Journal of neurosurgery 123, 1202-1208.

69. Welch, R.D., Ayaz, S.I., Lewis, L.M., Unden, J., Chen, J.Y., Mika, V.H., Saville, B., Tyndall, J.A., Nash, M., Buki, A., Barzo, P., Hack, D., Tortella, F.C., Schmid, K., Hayes, R.L., Vossough, A., Sweriduk, S.T. and Bazarian, J.J. (2016). Ability of Serum Glial Fibrillary Acidic Protein, Ubiquitin C-Terminal Hydrolase-L1, and S100B To Differentiate Normal and Abnormal Head Computed Tomography Findings in Patients with Suspected Mild or Moderate Traumatic Brain Injury. Journal of neurotrauma 33, 203-214.

70. Wolf, H., Frantal, S., Pajenda, G.S., Salameh, O., Widhalm, H., Hajdu, S. and Sarahrudi, K. (2013). Predictive value of neuromarkers supported by a set of clinical criteria in patients with mild traumatic brain injury: S100B protein and neuron-specific enolase on trial: clinical article. Journal of neurosurgery 118, 1298-1303.

71. Zongo, D., Ribereau-Gayon, R., Masson, F., Laborey, M., Contrand, B., Salmi, L.R., Montaudon, D., Beaudeux, J.L., Meurin, A., Dousset, V., Loiseau, H. and Lagarde, E. (2012). S100-B protein as a screening tool for the early assessment of minor head injury. Annals of emergency medicine 59, 209-218.

72. Babcock, L., Byczkowski, T., Wade, S.L., Ho, M. and Bazarian, J.J. (2013). Inability of S100B to predict postconcussion syndrome in children who present to the emergency department with mild traumatic brain injury: a brief report. Pediatric emergency care 29, 458-461.

73. Bazarian, J.J., Zemlan, F.P., Mookerjee, S. and Stigbrand, T. (2006). Serum S-100B and cleaved-tau are poor predictors of long-term outcome after mild traumatic brain injury. Brain injury 20, 759-765.

74. Chen, K.Y., Tsai, T.Y., Chang, C.F., Tsai, Y.R., Ou, J.C., Ma, H.P., Tsai, S.H., Chiu, W.T., Lin, J.W., Liao, K.H., Lin, C.M., Wu, J.C. and Chiang, Y.H. (2015). Worsening of dizziness impairment is associated with BMX level in patients after mild traumatic brain injury. Journal of neurotrauma.

75. de Boussard, C.N., Lundin, A., Karlstedt, D., Edman, G., Bartfai, A. and Borg, J. (2005). S100 and cognitive impairment after mild traumatic brain injury. Journal of rehabilitation medicine 37, 53-57.

76. Li, M., Lin, Y.P., Chen, J.L., Li, H., Jiang, R.C. and Zhang, J.N. (2015). Role of regulatory T cell in clinical outcome of traumatic brain injury. Chin Med J (Engl) 128, 1072-1078.

77. Ma, M., Lindsell, C.J., Rosenberry, C.M., Shaw, G.J. and Zemlan, F.P. (2008). Serum cleaved tau does not predict postconcussion syndrome after mild traumatic brain injury. The American journal of emergency medicine 26, 763-768.

78. Mannix, R., Eisenberg, M., Berry, M., Meehan, W.P., 3rd and Hayes, R.L. (2014). Serum biomarkers predict acute symptom burden in children after concussion: a preliminary study. Journal of neurotrauma 31, 1072-1075.

79. Rothoerl, R.D., Woertgen, C. and Brawanski, A. (2000). S-100 serum levels and outcome after severe head injury. Acta neurochirurgica. Supplement 76, 97-100.

80. Ryb, G.E., Dischinger, P.C., Auman, K.M., Kufera, J.A., Cooper, C.C., Mackenzie, C.F. and Kane, R.L. (2014). S-100beta does not predict outcome after mild traumatic brain injury. Brain injury 28, 1430-1435.

81. Savola, O., Pyhtinen, J., Leino, T.K., Siitonen, S., Niemela, O. and Hillbom, M. (2004). Effects of head and extracranial injuries on serum protein S100B levels in trauma patients. The Journal of trauma 56, 1229-1234; discussion 1234.

82. Shahim, P., Zetterberg, H., Tegner, Y. and Blennow, K. (2017). Serum neurofilament light as a biomarker for mild traumatic brain injury in contact sports. Neurology 88, 1788-1794.

83. Siman, R., Giovannone, N., Hanten, G., Wilde, E.A., McCauley, S.R., Hunter, J.V., Li, X., Levin, H.S. and Smith, D.H. (2013). Evidence That the Blood Biomarker SNTF Predicts Brain Imaging Changes and Persistent Cognitive Dysfunction in Mild TBI Patients. Frontiers in neurology 4, 190.

84. Stranjalis, G., Korfias, S., Papapetrou, C., Kouyialis, A., Boviatsis, E., Psachoulia, C. and Sakas, D.E. (2004). Elevated serum S-100B protein as a predictor of failure to short-term return to work or activities after mild head injury. Journal of neurotrauma 21, 1070-1075.

85. Su, S.H., Xu, W., Li, M., Zhang, L., Wu, Y.F., Yu, F. and Hai, J. (2014). Elevated C-reactive protein levels may be a predictor of persistent unfavourable symptoms in patients with mild traumatic brain injury: a preliminary study. Brain, behavior, and immunity 38, 111-117.

86. Topolovec-Vranic, J., Pollmann-Mudryj, M.A., Ouchterlony, D., Klein, D., Spence, J., Romaschin, A., Rhind, S., Tien, H.C. and Baker, A.J. (2011). The value of serum biomarkers in prediction models of outcome after mild traumatic brain injury. The Journal of trauma 71, S478-486.

87. Wang, H.C., Wang, P.M., Lin, Y.J., Kwan, A.L., Lin, W.C., Tsai, N.W., Cheng, B.C., Chang, W.N., Su, B.Y., Kung, C.T. and Lu, C.H. (2013). Serum adhesion molecules, outcome and neuro-psychological function in acute traumatic brain injury patients. Clinica chimica acta; international journal of clinical chemistry 423, 122-129.

88. Wilkinson, A.A., Simic, N., Frndova, H., Taylor, M.J., Choong, K., Fraser, D., Campbell, C., Dhanani, S., Kuehn, S., Beauchamp, M.H., Farrell, C., Anderson, V., Guerguerian, A.-M., Dennis, M., Schachar, R. and Hutchison, J.S. (2016). Serum Biomarkers Help Predict Attention Problems in Critically Ill Children With Traumatic Brain Injury. Pediatric Critical Care Medicine 17, 638-648.

89. Xu, Z., Lv, X.A., Wang, J.W., Chen, Z.P. and Qiu, H.S. (2014). Predictive value of early decreased plasma ghrelin level for three-month cognitive deterioration in patients with mild traumatic brain injury. Peptides 54, 180-185.

90. Bandyopadhyay, S., Hennes, H., Gorelick, M.H., Wells, R.G. and Walsh-Kelly, C.M. (2005). Serum neuron-specific enolase as a predictor of short-term outcome in children with closed traumatic brain injury. Academic emergency medicine : official journal of the Society for Academic Emergency Medicine 12, 732-738.

91. Barton, D.J., Kumar, R.G., McCullough, E.H., Galang, G., Arenth, P.M., Berga, S.L. and Wagner, A.K. (2016). Persistent Hypogonadotropic Hypogonadism in Men After Severe Traumatic Brain Injury. Journal of Head Trauma Rehabilitation 31, 277-287.

92. Bjugstad, K.B., Rael, L.T., Levy, S., Carrick, M., Mains, C.W., Slone, D.S. and Bar-Or, D. (2016). Oxidation-Reduction Potential as a Biomarker for Severity and Acute Outcome in Traumatic Brain Injury. Oxidative Medicine and Cellular Longevity 2016, 1-9.

93. Brophy, G.M., Mondello, S., Papa, L., Robicsek, S.A., Gabrielli, A., Tepas, J., 3rd, Buki, A., Robertson, C., Tortella, F.C., Hayes, R.L. and Wang, K.K. (2011). Biokinetic analysis of ubiquitin C-terminal hydrolase-L1 (UCH-L1) in severe traumatic brain injury patient biofluids. Journal of neurotrauma 28, 861-870.

94. Campello Yurgel, V., Ikuta, N., Brondani da Rocha, A., Lunge, V.R., Fett Schneider, R., Kazantzi Fonseca, A.S., Grivicich, I., Zanoni, C. and Regner, A. (2007). Role of plasma DNA as a predictive marker of fatal outcome following severe head injury in males. Journal of neurotrauma 24, 1172-1181.

95. Chabok, S.Y., Moghadam, A.D., Saneei, Z., Amlashi, F.G., Leili, E.K. and Amiri, Z.M. (2012). Neuron-specific enolase and S100BB as outcome predictors in severe diffuse axonal injury. The journal of trauma and acute care surgery 72, 1654-1657.

96. Chen, H., Cao, H.-L., Chen, S.-W., Guo, Y., Gao, W.-W., Tian, H.-L. and Xue, L.-X. (2015). Neuroglobin and Nogo-a as biomarkers for the severity and prognosis of traumatic brain injury. Biomarkers : biochemical indicators of exposure, response, and susceptibility to chemicals 20, 495-501.

97. Chen, Q.-H., Lin, D., Zhou, J. and Deng, G. (2016). Role of signal peptide-Cub-Egf domain-containing protein-1 in serum as a predictive biomarker of outcome after severe traumatic brain injury. Clinica Chimica Acta 456, 63-66.

98. Chiaretti, A., Genovese, O., Aloe, L., Antonelli, A., Piastra, M., Polidori, G. and Di Rocco, C. (2005). Interleukin 1beta and interleukin 6 relationship with paediatric head trauma severity and outcome. Child's nervous system : ChNS : official journal of the International Society for Pediatric Neurosurgery 21, 185-193; discussion 194.

99. da Rocha, A.B., Zanoni, C., de Freitas, G.R., Andre, C., Himelfarb, S., Schneider, R.F., Grivicich, I., Borges, L., Schwartsmann, G., Kaufmann, M. and Regner, A. (2005). Serum Hsp70 as an early predictor of fatal outcome after severe traumatic brain injury in males. Journal of neurotrauma 22, 966-977.

100. da Rocha, A.B., Schneider, R.F., de Freitas, G.R., Andre, C., Grivicich, I., Zanoni, C., Fossa, A., Gehrke, J.T., Pereira Jotz, G., Kaufmann, M., Simon, D. and Regner, A. (2006). Role of serum S100B as a predictive marker of fatal outcome following isolated severe head injury or multitrauma in males. Clinical chemistry and laboratory medicine : CCLM / FESCC 44, 1234-1242.

101. Dash, P.K., Redell, J.B., Hergenroeder, G., Zhao, J., Clifton, G.L. and Moore, A. (2010). Serum ceruloplasmin and copper are early biomarkers for traumatic brain injury-associated elevated intracranial pressure. Journal of neuroscience research 88, 1719-1726.

102. De Oliveira, C.O., Reimer, A.G., Da Rocha, A.B., Grivicich, I., Schneider, R.F., Roisenberg, I., Regner, A. and Simon, D. (2007). Plasma von Willebrand factor levels correlate with clinical outcome of severe traumatic brain injury. Journal of neurotrauma 24, 1331-1338.

103. DeFazio, M.V., Rammo, R.A., Robles, J.R., Bramlett, H.M., Dietrich, W.D. and Bullock, M.R. (2014). The potential utility of blood-derived biochemical markers as indicators of early clinical trends following severe traumatic brain injury. World neurosurgery 81, 151-158.

104. Di Battista, A.P., Buonora, J.E., Rhind, S.G., Hutchison, M.G., Baker, A.J., Rizoli, S.B., Diaz-Arrastia, R. and Mueller, G.P. (2015). Blood Biomarkers in Moderate-To-Severe Traumatic Brain Injury: Potential Utility of a Multi-Marker Approach in Characterizing Outcome. Frontiers in neurology 6.

105. Dong, X.Q., Huang, M., Yang, S.B., Yu, W.H. and Zhang, Z.Y. (2011). Copeptin is associated with mortality in patients with traumatic brain injury. The Journal of trauma 71, 1194-1198.

106. Dong, X.Q., Yu, W.H., Du, Q., Wang, H., Zhu, Q., Yang, D.B., Che, Z.H., Shen, Y.F. and Jiang, L. (2017). Serum periostin concentrations and outcomes after severe traumatic brain injury. Clinica chimica acta; international journal of clinical chemistry 471, 298-303.

107. Dong, X.Q., Yu, W.H., Zhang, Z.Y., Yang, D.B., Du, Q., Wang, H., Shen, Y.F., Jiang, L., Che, Z.H. and Zhu, Q. (2017). Serum thioredoxin and in-hospital major adverse events after traumatic brain injury. Clinica chimica acta; international journal of clinical chemistry 469, 75-80.

108. Egea-Guerrero, J.J., Murillo-Cabezas, F., Gordillo-Escobar, E., Rodriguez-Rodriguez, A., Enamorado-Enamorado, J., Revuelto-Rey, J., Pacheco-Sanchez, M., Leon-Justel, A., Dominguez-Roldan, J.M. and Vilches-Arenas, A. (2013). S100B protein may detect brain death development after severe traumatic brain injury. Journal of neurotrauma 30, 1762-1769.

109. Feng, M.J., Ning, W.B., Wang, W., Lv, Z.H., Liu, X.B., Zhu, Y., Gao, W., Jin, H.Z. and Gao, S.S. (2018). Serum S100A12 as a prognostic biomarker of severe traumatic brain injury. Clinica chimica acta; international journal of clinical chemistry 480, 84-91.

110. Foaud, H.M., Labib, J.R., Metwally, H.G. and El-Twab, K.M. (2014). Plasma D-dimer as a Prognostic Marker in ICU Admitted Egyptian Children with Traumatic Brain Injury. Journal of clinical and diagnostic research : JCDR 8, PC01-06.

111. Fraser, D.D., Close, T.E., Rose, K.L., Ward, R., Mehl, M., Farrell, C., Lacroix, J., Creery, D., Kesselman, M., Stanimirovic, D. and Hutchison, J.S. (2011). Severe traumatic brain injury in children elevates glial fibrillary acidic protein in cerebrospinal fluid and serum. Pediatric critical care medicine : a journal of the Society of Critical Care Medicine and the World Federation of Pediatric Intensive and Critical Care Societies 12, 319-324.

112. Gonzclez-Mao, M.C., Reparaz-Andrade, A., Del Campo-Perez, V., Alvarez-Garcia, E., Vara-Perez, C. and Andrade-Olivie, M.A. (2011). Model predicting survival/exitus after traumatic brain injury: biomarker S100B 24h. Clinical laboratory 57, 587-597.

113. Jin, Y., Li, B.Y., Qiu, L.L., Ling, Y.R. and Bai, Z.Q. (2012). Decreased plasma gelsolin is associated with 1-year outcome in patients with traumatic brain injury. Journal of critical care 27, 527 e521-526.

114. Karri, J., Cardenas, J.C., Matijevic, N., Wang, Y.W., Choi, S., Zhu, L., Cotton, B.A., Kitagawa, R., Holcomb, J.B. and Wade, C.E. (2017). Early Fibrinolysis Associated with Hemorrhagic Progression Following Traumatic Brain Injury. Shock 48, 644-650.

115. Lei, J., Gao, G., Feng, J., Jin, Y., Wang, C., Mao, Q. and Jiang, J. (2015). Glial fibrillary acidic protein as a biomarker in severe traumatic brain injury patients: a prospective cohort study. Critical Care 19.

116. Lee, J.Y., Lee, C.Y., Kim, H.R., Lee, C.-H., Kim, H.W. and Kim, J.H. (2015). A Role of Serum-Based Neuronal and Glial Markers as Potential Predictors for Distinguishing Severity and Related Outcomes in Traumatic Brain Injury. Journal of Korean Neurosurgical Society 58, 93.

117. Lee, D.H., Lee, B.K., Noh, S.M. and Cho, Y.S. (2018). High fibrin/fibrinogen degradation product to fibrinogen ratio is associated with 28-day mortality and massive transfusion in severe trauma. European journal of trauma and emergency surgery : official publication of the European Trauma Society 44, 291-298.

118. Li, N., Shen, J.K., Zhao, W.G., Cai, Y., Li, Y.F. and Zhan, S.K. (2004). S-100B and neuron specific enolase in outcome prediction of severe head injury. Chinese journal of traumatology = Zhonghua chuang shang za zhi / Chinese Medical Association 7, 156-158.

119. Liliang, P.C., Liang, C.L., Weng, H.C., Lu, K., Wang, K.W., Chen, H.J. and Chuang, J.H. (2010). Tau proteins in serum predict outcome after severe traumatic brain injury. The Journal of surgical research 160, 302-307.

120. Lin, C., Wang, N., Shen, Z.P. and Zhao, Z.Y. (2013). Plasma copeptin concentration and outcome after pediatric traumatic brain injury. Peptides 42, 43-47.

121. Lin, C., Huang, S.J., Wang, N. and Shen, Z.P. (2012). Relationship between plasma leptin levels and clinical outcomes of pediatric traumatic brain injury. Peptides 35, 166-171.

122. Lorente, L., Martin, M.M., Lopez, P., Ramos, L., Blanquer, J., Caceres, J.J., Sole-Violan, J., Solera, J., Cabrera, J., Argueso, M., Ortiz, R., Mora, M.L., Lubillo, S., Jimenez, A., Borreguero-Leon, J.M., Gonzalez, A., Orbe, J., Rodriguez, J.A. and Paramo, J.A. (2014). Association between serum tissue inhibitor of matrix metalloproteinase-1 levels and mortality in patients with severe brain trauma injury. PloS one 9, e94370.

123. Lorente, L., Martín, M.M., Almeida, T., Hernández, M., Ramos, L., Argueso, M., Cáceres, J.J., Solé-Violán, J. and Jiménez, A. (2015). Serum substance P levels are associated with severity and mortality in patients with severe traumatic brain injury. Critical Care 19.

124. Lorente, L., Martín, M.M., Abreu-González, P., Ramos, L., Argueso, M., Cáceres, J.J., Solé-Violán, J., Lorenzo, J.M., Molina, I. and Jiménez, A. (2015). Association between Serum Malondialdehyde Levels and Mortality in Patients with Severe Brain Trauma Injury. Journal of neurotrauma 32, 1-6.

125. Lorente, L., Martin, M.M., Gonzalez-Rivero, A.F., Argueso, M., Ramos, L., Sole-Violan, J., Caceres, J.J., Jimenez, A. and Borreguero-Leon, J.M. (2015). Serum levels of caspase-cleaved cytokeratin-18 in patients with severe traumatic brain injury are associated with mortality: a pilot study. PloS one 10, e0121739.

126. Lorente, L., Martín, M.M., Almeida, T., Abreu-González, P., Ramos, L., Argueso, M., Riaño-Ruiz, M., Solé-Violán, J. and Jiménez, A. (2015). Total antioxidant capacity is associated with mortality of patients with severe traumatic brain injury. BMC neurology 15.

127. Macher, H., Egea-Guerrero, J.J., Revuelto-Rey, J., Gordillo-Escobar, E., Enamorado-Enamorado, J., Boza, A., Rodriguez, A., Molinero, P., Guerrero, J.M., Dominguez-Roldan, J.M., Murillo-Cabezas, F. and Rubio, A. (2012). Role of early cell-free DNA levels decrease as a predictive marker of fatal outcome after severe traumatic brain injury. Clinica chimica acta; international journal of clinical chemistry 414, 12-17.

128. Meric, E., Gunduz, A., Turedi, S., Cakir, E. and Yandi, M. (2010). The prognostic value of neuron-specific enolase in head trauma patients. The Journal of emergency medicine 38, 297-301.

129. Mondello, S., Papa, L., Buki, A., Bullock, M.R., Czeiter, E., Tortella, F.C., Wang, K.K. and Hayes, R.L. (2011). Neuronal and glial markers are differently associated with computed tomography findings and outcome in patients with severe traumatic brain injury: a case control study. Crit Care 15, R156.

130. Mortberg, E., Zetterberg, H., Nordmark, J., Blennow, K., Catry, C., Decraemer, H., Vanmechelen, E. and Rubertsson, S. (2011). Plasma tau protein in comatose patients after cardiac arrest treated with therapeutic hypothermia. Acta anaesthesiologica Scandinavica 55, 1132-1138.

131. Murillo-Cabezas, F., Munoz-Sanchez, M.A., Rincon-Ferrari, M.D., Martin-Rodriguez, J.F., Amaya-Villar, R., Garcia-Gomez, S. and Leon-Carrion, J. (2010). The prognostic value of the temporal course of S100beta protein in post-acute severe brain injury: A prospective and observational study. Brain injury 24, 609-619.

132. Mussack, T., Biberthaler, P., Kanz, K.G., Wiedemann, E., Gippner-Steppert, C., Mutschler, W. and Jochum, M. (2002). Serum S-100B and interleukin-8 as predictive markers for comparative neurologic outcome analysis of patients after cardiac arrest and severe traumatic brain injury. Critical care medicine 30, 2669-2674.

133. Nylen, K., Ost, M., Csajbok, L.Z., Nilsson, I., Blennow, K., Nellgard, B. and Rosengren, L. (2006). Increased serum-GFAP in patients with severe traumatic brain injury is related to outcome. Journal of the neurological sciences 240, 85-91.

134. Nylen, K., Ost, M., Csajbok, L.Z., Nilsson, I., Hall, C., Blennow, K., Nellgard, B. and Rosengren, L. (2008). Serum levels of S100B, S100A1B and S100BB are all related to outcome after severe traumatic brain injury. Acta neurochirurgica 150, 221-227; discussion 227.

135. Olivecrona, Z., Bobinski, L. and Koskinen, L.O. (2015). Association of ICP, CPP, CT findings and S-100B and NSE in severe traumatic head injury. Prognostic value of the biomarkers. Brain injury 29, 446-454.

136. Pan, J.-W., Gao, X.-W., Jiang, H., Li, Y.-F., Xiao, F. and Zhan, R.-Y. (2015). Low serum ficolin-3 levels are associated with severity and poor outcome in traumatic brain injury. Journal of neuroinflammation 12.

137. Pelinka, L.E., Toegel, E., Mauritz, W. and Redl, H. (2003). Serum S 100 B: a marker of brain damage in traumatic brain injury with and without multiple trauma. Shock 19, 195-200.

138. Petzold, A., Green, A.J., Keir, G., Fairley, S., Kitchen, N., Smith, M. and Thompson, E.J. (2002). Role of serum S100B as an early predictor of high intracranial pressure and mortality in brain injury: a pilot study. Critical care medicine 30, 2705-2710.

139. Pleines, U.E., Stover, J.F., Kossmann, T., Trentz, O. and Morganti-Kossmann, M.C. (1998). Soluble ICAM-1 in CSF coincides with the extent of cerebral damage in patients with severe traumatic brain injury. Journal of neurotrauma 15, 399-409.

140. Raabe, A., Grolms, C., Keller, M., Dohnert, J., Sorge, O. and Seifert, V. (1998). Correlation of computed tomography findings and serum brain damage markers following severe head injury. Acta neurochirurgica 140, 787-791; discussion 791-782.

141. Raabe, A., Grolms, C., Sorge, O., Zimmermann, M. and Seifert, V. (1999). Serum S-100B protein in severe head injury. Neurosurgery 45, 477-483.

142. Raabe, A., Grolms, C. and Seifert, V. (1999). Serum markers of brain damage and outcome prediction in patients after severe head injury. British journal of neurosurgery 13, 56-59.

143. Raheja, A., Sinha, S., Samson, N., Bhoi, S., Subramanian, A., Sharma, P. and Sharma, B.S. (2016). Serum biomarkers as predictors of long-term outcome in severe traumatic brain injury: analysis from a randomized placebo-controlled Phase II clinical trial. Journal of neurosurgery 125, 631-641.

144. Rainey, T., Lesko, M., Sacho, R., Lecky, F. and Childs, C. (2009). Predicting outcome after severe traumatic brain injury using the serum S100B biomarker: results using a single (24h) time-point. Resuscitation 80, 341-345.

145. Randall, J., Mortberg, E., Provuncher, G.K., Fournier, D.R., Duffy, D.C., Rubertsson, S., Blennow, K., Zetterberg, H. and Wilson, D.H. (2013). Tau proteins in serum predict neurological outcome after hypoxic brain injury from cardiac arrest: results of a pilot study. Resuscitation 84, 351-356.

146. Rodriguez-Rodriguez, A., Egea-Guerrero, J.J., Leon-Justel, A., Gordillo-Escobar, E., Revuelto-Rey, J., Vilches-Arenas, A., Carrillo-Vico, A., Dominguez-Roldan, J.M., Murillo-Cabezas, F. and Guerrero, J.M. (2012). Role of S100B protein in urine and serum as an early predictor of mortality after severe traumatic brain injury in adults. Clinica chimica acta; international journal of clinical chemistry 414, 228-233.

147. Rodríguez-Rodríguez, A., Egea-Guerrero, J.J., Gordillo-Escobar, E., Enamorado-Enamorado, J., Hernández-García, C., Ruiz de Azúa-López, Z., Vilches-Arenas, Á., Guerrero, J.M. and Murillo-Cabezas, F. (2016). S100B and Neuron-Specific Enolase as mortality predictors in patients with severe traumatic brain injury. Neurological research 38, 130-137.

148. Sadaka, F., Doctors, N., Pearson, T., Snyders, B. and O'Brien, J. (2018). Does Red Cell Distribution Width Predict Outcome in Traumatic Brain Injury: Comparison to Corticosteroid Randomization After Significant Head Injury. Journal of clinical medicine research 10, 9-12.

149. Schneider Soares, F.M., Menezes de Souza, N., Liborio Schwarzbold, M., Paim Diaz, A., Costa Nunes, J., Hohl, A., Nunes Abreu da Silva, P., Vieira, J., Lisboa de Souza, R., More Bertotti, M., Schoder Prediger, R.D., Neves Linhares, M., Bafica, A. and Walz, R. (2012). Interleukin-10 is an independent biomarker of severe traumatic brain injury prognosis. Neuroimmunomodulation 19, 377-385.

150. Shahim, P., Gren, M., Liman, V., Andreasson, U., Norgren, N., Tegner, Y., Mattsson, N., Andreasen, N., Ost, M., Zetterberg, H., Nellgard, B. and Blennow, K. (2016). Serum neurofilament light protein predicts clinical outcome in traumatic brain injury. Sci Rep 6, 36791.

151. Shallwani, H., Waqas, M., Waheed, S., Siddiqui, M., Froz, A. and Bari, M.E. (2015). Does base deficit predict mortality in patients with severe traumatic brain injury? International Journal of Surgery 22, 125-130.

152. Shaw, G.J., Jauch, E.C. and Zemlan, F.P. (2002). Serum cleaved tau protein levels and clinical outcome in adult patients with closed head injury. Annals of emergency medicine 39, 254-257.

153. Shen, L.J., Yang, S.B., Lv, Q.W., Zhang, G.H., Zhou, J., Guo, M., Huang, H.B., Li, Z. and Yang, C.S. (2014). High plasma adiponectin levels in patients with severe traumatic brain injury. Clinica chimica acta; international journal of clinical chemistry 427, 37-41.

154. Shen, Y.-F., Yu, W.-H., Dong, X.-Q., Du, Q., Yang, D.-B., Wu, G.-Q., Zhang, Z.-Y., Wang, H. and Jiang, L. (2016). The change of plasma galectin-3 concentrations after traumatic brain injury. Clinica Chimica Acta 456, 75-80.

155. Simon, D., Nicol, J.M., Sabino da Silva, S., Graziottin, C., Silveira, P.C., Ikuta, N. and Regner, A. (2015). Serum ferritin correlates with Glasgow coma scale scores and fatal outcome after severe traumatic brain injury. Brain injury 29, 612-617.

156. Simon, D., Evaldt, J., Nabinger, D.D., Fontana, M.F., Klein, M.G., do Amaral Gomes, J. and Regner, A. (2017). Plasma matrix metalloproteinase-9 levels predict intensive care unit mortality early after severe traumatic brain injury. Brain injury 31, 390-395.

157. Spinella, P.C., Dominguez, T., Drott, H.R., Huh, J., McCormick, L., Rajendra, A., Argon, J., McIntosh, T. and Helfaer, M. (2003). S-100beta protein-serum levels in healthy children and its association with outcome in pediatric traumatic brain injury. Critical care medicine 31, 939-945.

158. Takala, R.S.K., Posti, J.P., Runtti, H., Newcombe, V.F., Outtrim, J., Katila, A.J., Frantzén, J., Ala-Seppälä, H., Kyllönen, A., Maanpää, H.-R., Tallus, J., Hossain, M.I., Coles, J.P., Hutchinson, P., van Gils, M., Menon, D.K. and Tenovuo, O. (2016). Glial Fibrillary Acidic Protein and Ubiquitin C-Terminal Hydrolase-L1 as Outcome Predictors in Traumatic Brain Injury. World neurosurgery 87, 8-20.

159. Vos, P.E., Lamers, K.J., Hendriks, J.C., van Haaren, M., Beems, T., Zimmerman, C., van Geel, W., de Reus, H., Biert, J. and Verbeek, M.M. (2004). Glial and neuronal proteins in serum predict outcome after severe traumatic brain injury. Neurology 62, 1303-1310.

160. Vos, P.E., Jacobs, B., Andriessen, T.M., Lamers, K.J., Borm, G.F., Beems, T., Edwards, M., Rosmalen, C.F. and Vissers, J.L. (2010). GFAP and S100B are biomarkers of traumatic brain injury: an observational cohort study. Neurology 75, 1786-1793.

161. Walder, B., Robin, X., Rebetez, M.M., Copin, J.C., Gasche, Y., Sanchez, J.C. and Turck, N. (2013). The prognostic significance of the serum biomarker heart-fatty acidic binding protein in comparison with s100b in severe traumatic brain injury. Journal of neurotrauma 30, 1631-1637.

162. Wang, H.-C., Lin, Y.-J., Shih, F.-Y., Chang, H.-W., Su, Y.-J., Cheng, B.-C., Su, C.-M., Tsai, N.-W., Chang, Y.-T., Kwan, A.-L. and Lu, C.-H. (2016). The Role of Serial Oxidative Stress Levels in Acute Traumatic Brain Injury and as Predictors of Outcome. World neurosurgery 87, 463-470.

163. Wang, J., Li, J., Han, L., Guo, S., Wang, L., Xiong, Z., Chen, Z., Chen, W. and Liang, J. (2016). Serum τï¿½protein as a potential biomarker in the assessment of traumatic brain injury. Experimental and Therapeutic Medicine.

164. Wang, J.L., Jin, G.L., Yuan, Z.G., Yu, X.B., Li, J.Q., Qiu, T.L. and Dai, R.X. (2016). Plasma thrombospondin-1 and clinical outcomes in traumatic brain injury. Acta neurologica Scandinavica 134, 189-196.

165. Wang, K.Y., Yu, G.F., Zhang, Z.Y., Huang, Q. and Dong, X.Q. (2012). Plasma high-mobility group box 1 levels and prediction of outcome in patients with traumatic brain injury. Clinica chimica acta; international journal of clinical chemistry 413, 1737-1741.

166. Woertgen, C., Rothoerl, R.D., Metz, C. and Brawanski, A. (1999). Comparison of clinical, radiologic, and serum marker as prognostic factors after severe head injury. The Journal of trauma 47, 1126-1130.

167. Woertgen, C., Rothoerl, R.D. and Brawanski, A. (2002). Early S-100B serum level correlates to quality of life in patients after severe head injury. Brain injury 16, 807-816.

168. Woiciechowsky, C., Schoning, B., Cobanov, J., Lanksch, W.R., Volk, H.D. and Docke, W.D. (2002). Early IL-6 plasma concentrations correlate with severity of brain injury and pneumonia in brain-injured patients. The Journal of trauma 52, 339-345.

169. Wu, G.-Q., Chou, X.-M., Ji, W.-J., Yang, X.-G., Lan, L.-X., Sheng, Y.-J., Shen, Y.-F., Li, J.-R., Huang, G.-Z., Yu, W.-H., Dong, X.-Q., Du, Q., Yang, D.-B., Zhang, Z.-Y., Wang, H., Shen, Y.-F. and Jiang, L. (2016). The prognostic value of plasma nesfatin-1 concentrations in patients with traumatic brain injury. Clinica Chimica Acta 458, 124-128.

170. Xu, J.F., Liu, W.G., Dong, X.Q., Yang, S.B. and Fan, J. (2011). Change in Plasma Gelsolin Level After Traumatic Brain Injury. The Journal of trauma.

171. Yamazaki, Y., Yada, K., Morii, S., Kitahara, T. and Ohwada, T. (1995). Diagnostic significance of serum neuron-specific enolase and myelin basic protein assay in patients with acute head injury. Surgical neurology 43, 267-270; discussion 270-261.

172. Yang, D.B., Yu, W.H., Dong, X.Q., Du, Q., Shen, Y.F., Zhang, Z.Y., Zhu, Q., Che, Z.H., Liu, Q.J., Wang, H., Jiang, L. and Du, Y.F. (2014). Plasma copeptin level predicts acute traumatic coagulopathy and progressive hemorrhagic injury after traumatic brain injury. Peptides 58, 26-29.

173. Yang, D.B., Yu, W.H., Dong, X.Q., Zhang, Z.Y., Du, Q., Zhu, Q., Che, Z.H., Wang, H., Shen, Y.F. and Jiang, L. (2017). Serum macrophage migration inhibitory factor concentrations correlate with prognosis of traumatic brain injury. Clinica chimica acta; international journal of clinical chemistry 469, 99-104.

174. Yu, L., Wu, X., Wang, H., Long, D., Yang, J. and Zhang, Y. (2014). Diagnostic and prognostic significance of suPAR in traumatic brain injury. Neurology India 62, 498-502.

175. Yu, W., Le, H.-W., Lu, Y.-G., Hu, J.-A., Yu, J.-B., Wang, M. and Shen, W. (2015). High levels of serum mannose-binding lectins are associated with the severity and clinical outcomes of severe traumatic brain injury. Clinica Chimica Acta 451, 111-116.

176. Zhang, B., Zhang, Q.-H., Li, A.-M., He, S.-L., Yao, X.-D., Zhu, J., Zhang, Z.-W., Sheng, Z.-Y. and Yao, Y.-M. (2015). Serum Total Cholinesterase Activity on Admission Is Associated with Disease Severity and Outcome in Patients with Traumatic Brain Injury. PloS one 10, e0129082.

177. Zhang, B. and Zhao, J. (2015). Red blood cell distribution width as a prognostic biomarker for mortality in traumatic brain injury. International journal of clinical and experimental medicine 8, 19172-19175.

178. Zhang, Z.Y., Zhang, L.X., Dong, X.Q., Yu, W.H., Du, Q., Yang, D.B., Shen, Y.F., Wang, H., Zhu, Q., Che, Z.H., Liu, Q.J., Jiang, L. and Du, Y.F. (2014). Comparison of the performances of copeptin and multiple biomarkers in long-term prognosis of severe traumatic brain injury. Peptides 60, 13-17.

179. Zhao, Y.Y., Lou, L., Yang, K.C., Wang, H.B., Xu, Y., Lu, G. and He, H.Y. (2017). Correlation of tenascin-C concentrations in serum with outcome of traumatic brain injury in humans. Clinica chimica acta; international journal of clinical chemistry 472, 46-50.

180. Zurek, J., Bartlova, L. and Fedora, M. (2011). Hyperphosphorylated neurofilament NF-H as a predictor of mortality after brain injury in children. Brain injury 25, 221-226.
