# Supplementary material for: Blood Biomarkers for Traumatic Brain Injury: A Quantitative Assessment of Diagnostic and Prognostic Accuracy
Source: Front Neurol. 2019 Apr 26;10:446. doi: 10.3389/fneur.2019.00446 (PMC6498532; doi:10.3389/fneur.2019.00446)
Supplement: Supplementary file 2 [file Data_Sheet_2.docx]

**Images used:**

Neuron Hand-tuned.svg by Quasar Jarosz. Licensed under the Creative Commons Attribution-ShareAlike 3.0 Unported license. <https://creativecommons.org/licenses/by-sa/3.0/>. Changes: added labels.

Microglia.jpeg by Servier Medical Art. Licensed under the Creative Commons Attribution 3.0 Unported license. <https://creativecommons.org/licenses/by/3.0/>. Changes: added labels.

Diagram of an astrocyte - a type of glial cell CRUK 029.svg by Cancer Research UK. Licensed under the Creative Commons Attribution-Share Alike 4.0 International license. <https://creativecommons.org/licenses/by-sa/4.0/deed.en>. Changes: added labels.

Blood vessels brain english.jpg by Armin Kübelbeck. Licensed under the Creative Commons Attribution 1.0 Generic license. <https://creativecommons.org/licenses/by/1.0/deed.en>. Changes: cropped sections and added labels.

Basal_Ganglia_and_Related_Structures.svg. Public Domain.

Liver.svg. Public Domain.
